# PABPN1 functions as a hub in the assembly of nuclear poly(A) domains that are essential for mouse oocyte development

**DOI:** 10.1126/sciadv.abn9016

**Published:** 2022-10-28

**Authors:** Xing-Xing Dai, Shuai-Bo Pi, Long-Wen Zhao, Yun-Wen Wu, Jing-Ling Shen, Song-Ying Zhang, Qian-Qian Sha, Heng-Yu Fan

**Affiliations:** ^1^ MOE Key Laboratory for Biosystems Homeostasis and Protection and Innovation Center for Cell Signaling Network, Life Sciences Institute, Zhejiang University, Hangzhou 310058, China.; ^2^ Institute of Life Sciences, College of Life and Environmental Sciences, Wenzhou University, Wenzhou 325035, China.; ^3^ Key Laboratory of Reproductive Dysfunction Management of Zhejiang Province, Sir Run Run Shaw Hospital, School of Medicine, Zhejiang University, Hangzhou, China.; ^4^ Fertility Preservation Laboratory, Reproductive Medicine Center, Guangdong Second Provincial General Hospital, 510317 Guangzhou, China.

## Abstract

Growing oocytes store a large amount of maternal mRNA to support the subsequent “maternal-zygotic transition” process. At present, it is not clear how the growing oocytes store and process the newly transcribed mRNA under physiological conditions. In this study, we report non–membrane-bound compartments, nuclear poly(A) domains (NPADs), as the hub for newly transcribed mRNA, in developing mouse oocytes. The RNA binding protein PABPN1 promotes the formation of NPAD through its N-terminal disordered domain and RNA-recognized motif by means of liquid phase separation. *Pabpn1*-null growing oocytes cannot form NPAD normally in vivo and have defects in stability of oocyte growing–related transcripts and formation of long 3′ untranslated region isoform transcripts. Ultimately, *Pabpn1^fl/fl^;Gdf9-Cre* mice are completely sterile with primary ovarian insufficiency. These results demonstrate that NPAD formed by the phase separation properties of PABPN1-mRNA are the hub of the newly transcribed mRNA and essential for the development of oocytes and female reproduction.

## Teaser

NPADs formed by the phase separation properties of PABPN1-mRNA are essential for the development of oocytes.

## INTRODUCTION

In all animals, large amounts of maternal mRNA are stored to support the “maternal-to-zygotic transition (MZT)” process after fertilization (*1*). Transcriptome sequencing results indicate that the maternal transcriptome changes drastically during MZT. In recent years, the sequencing technology to detect the polyadenylate [poly(A)] tails of mRNAs (Tail-seq) has gradually developed, revealing that the poly(A) tails of mRNAs change dynamically during the MZT process (*2*). However, the dynamics of the distribution and location of these poly(A)-tailed mRNAs (PA^+^) during oocyte growth and meiosis maturation have not been described under physiological conditions. The dynamic distribution of PA^+^, their formation, and the physiological significance remain unclear.

The stability of an mRNA is related to the poly(A) tail length of its transcript (*2*). Poly(A)-binding protein (PABP) is an RNA binding protein that can bind to the poly(A) tail of mRNA (*3*). There are two different PABPs in vertebrates: PABP in the nucleus, namely, PABPN1 and PABPN1L; and PABP in the cytoplasm, namely, PABPC1 and its homologous proteins (*4*, *5*). Recent studies have shown that *Pabpn1l* (also known as *Pabpn1*-like) of the PABP family is specifically and abundantly expressed in oocytes. PABPN1L acts as a poly(A)-binding adaptor for the mammalian MZT licensing factor BTG4 and regulates the clearance of maternal mRNA. *Pabpn1l-*knockout female mice have been shown to produce oocytes with normal morphology; however, embryonic development was found to be blocked at the one- to two-cell stage after fertilization (*6*). *Pabpn1* is a homologous gene of *Pabpn1l* that is widely expressed in various tissues and organs (*7*). However, it is not clear whether PABPN1 is expressed in growing and mature oocytes and has a physiological function that does not overlap with that of PABPN1L during this process.

PABPN1 participates in multiple cellular steps of mRNA metabolism, as revealed by in vitro biochemical studies and investigations in cultured cell lines (*8*). PABPN1 can bind to the proximal polyadenylation signal (PAS) in the 3′ untranslated region (3′UTR), thus preventing the binding of cleavage and polyadenylation specificity factor (CPSF) to PAS (*9*). PABPN1 stabilizes the docking of mRNA poly(A) polymerase (PAP) on mRNA 3′ ends and improves the polyadenylation activity of PAP (*10*). In addition, the combination of PABPN1 and poly(A) also controls the length of the mRNA poly(A) tail to 200 to 300 adenylate in the nucleus (*11*). In addition, PABPN1 participates in regulating the transport of mature mRNA from the nucleus to the cytoplasm (*8*).

The processing, transportation, and accumulation of maternal mRNA determine the developmental potential of oocytes, and these are the potential regulatory functions of PABPN1. *Pabpn1*-knockout embryos die in the early stages of development, further indicating that this gene has important functions in vivo. Therefore, it is necessary to study the relationship between the dynamic distribution of mRNA and PABPN1 in oocytes under physiological conditions and use oocyte-specific *Pabpn1-*knockout mice to explore the physiological significance of the dynamic distribution and processing of mRNA.

Many cellular compartments, including RNA-protein granules such as nucleoli, Cajal bodies, and promyelocytic leukemia nuclear bodies in the nucleus (*12*, *13*), as well as stress granules and germ granules in the cytoplasm, are non–membrane-bound compartments (*14*–*16*). Mounting evidence suggests that these membraneless compartments form via liquid-liquid condensation properties, which are driven by weak, multivalent interactions between proteins (containing intrinsically disordered domains) and nucleic acids (*16*–*19*). PABPN1 has an RNA binding sequence, RNA-recognized motif (RRM), with a high specific affinity to poly(A), and an N-terminal disordered domain (Nter). Whether PABPN1 promotes the formation of nuclear mRNA processing domains in the nucleus by means of condensation, to promote the processing of mRNA, remains to be studied.

In this study, we suggested that PABPN1 can promote the formation of nuclear poly(A) domains (NAPDs) in the nucleus and the processing of newly transcribed mRNAs by means of its condensation properties. We also constructed *Pabpn1* conditional-knockout mice and used them to confirm that NPADs cannot form normally in *Pabpn1*-null growing oocytes in vivo. Last, we proposed that the NPADs formed by the condensation properties of PABPN1-mRNA promote the formation of long 3′UTR transcripts, and this structure is essential for female reproduction.

## RESULTS

### PABPN1 and mRNAs with poly(A) tails (PA^+^) are dynamically compartmentalized in the nuclei of growing mouse oocytes

**Fig. 1.**
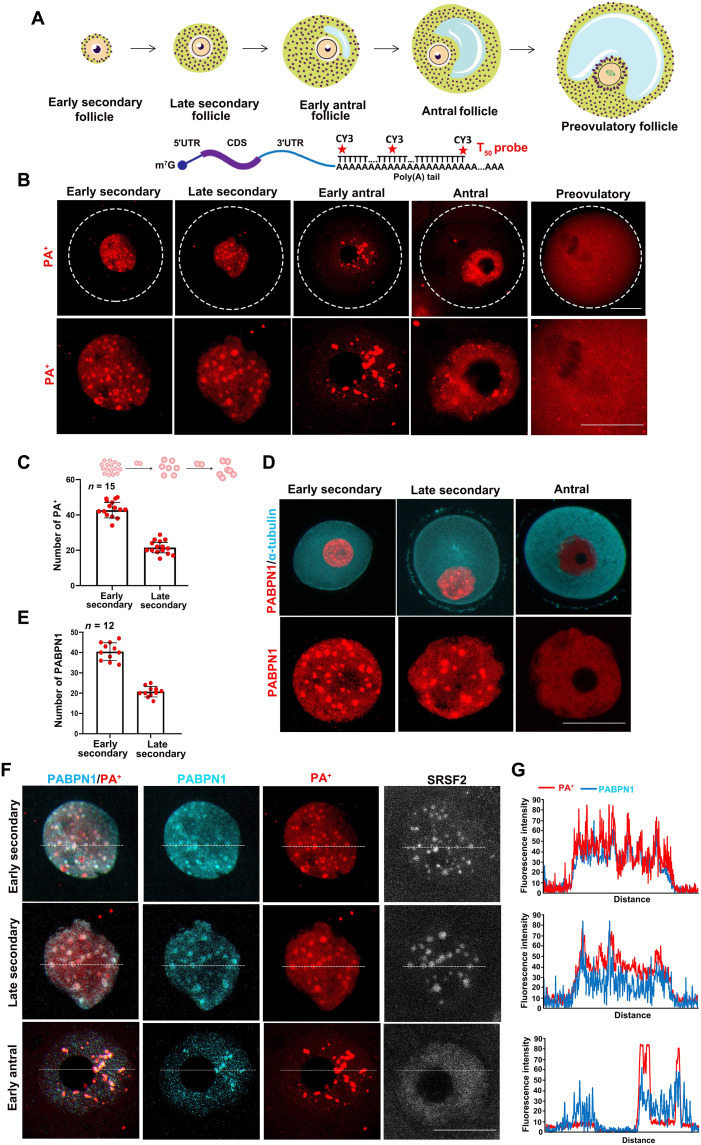
mRNA with poly(A) tails (PA^+^) and PABPN1 are colocalized in growing mouse oocytes. (**A**) Schematic diagram showing the collected oocytes at various stages of the follicles. The fluorescent probe, T_50_, recognizes mRNA with poly(A) tails. CDS, coding sequence. (**B**) FISH results showed that mRNAs with poly(A) tails (PA^+^) display dynamic nuclear compartmentalization in mouse oocytes, from the growing stages (early secondary follicle, late secondary follicle, early antral follicle, and antral follicle) to the meiotic maturation stage (preovulatory follicle). Scale bars, 20 μm. (**C**) Quantification of the number of PA^+^ condensates in growing oocytes from early secondary and late secondary follicle stages. (**D**) Immunofluorescence and confocal microscopy results showed the localization of PABPN1 in growing and fully grown oocytes. Scale bar, 20 μm. (**E**) Quantification of the number of PABPN1 condensates in growing oocytes from early secondary and late secondary follicles. (**F**) Immunofluorescence was used to detect the signals of PABPN1, PA^+^, and SRSF2 in the growing oocytes. Scale bar, 20 μm. (**G**) ImageJ was used to quantitatively analyze the distribution of PABPN1 and PA^+^ in (F), indicated in the figure by means of a white dotted line.

We detected the dynamic distribution of poly(A)-tailed mRNA (PA^+^) in mouse oocytes at different stages using a cyanine dye Cy3-labeled fluorescent poly(T) probe that recognizes mRNAs with poly(A) tails (T_50_) (Fig. 1A). The results of fluorescence in situ hybridization (FISH) assays showed that PA^+^ was distributed in multiple condensates in the nuclei of the growing oocytes (Fig. 1B). As the oocytes grew, the number of PA^+^ condensates decreased, while the volume in the nucleus increased (Fig. 1, B and C, and fig. S1A). In the fully grown oocytes within the large antral follicles, PA^+^ became more diffusely distributed in the nucleoplasm, as compared to that in the growing oocytes. During meiotic divisions, PA^+^ diffused into the cytoplasm (Fig. 1B).

PABPN1 is an RNA binding protein that can bind to the poly(A) tails of mRNA in the nucleus. The expression level of PABPN1 in growing oocytes was higher than that in fully grown oocytes (fig. S2, A and B). The results of immunofluorescence showed that PABPN1 had a similar distribution to PA^+^ in the oocyte nucleus (Fig. 1, D and E, and fig. S1B). Furthermore, PABPN1 colocalized with PA^+^ in the nuclei of the growing oocytes (Fig. 1, F and G).

The nucleus contains many membraneless domains. Serine/arginine-rich splicing factor 2 (SRSF2, also known as SC35) is a biomarker for nuclear speckles in somatic cells. This domain enriches factors related to transcription, mRNA processing, and transportation (*20*). Immunofluorescence results and linearized localization analysis showed that PABPN1, PA^+^, and SRSF2 colocalized in the nucleus of early growing oocytes corresponding to the secondary follicle stage (Fig. 1, F and G, and fig. S2, C and D). However, when the follicles grew to the early antral stage, PABPN1 and PA^+^ remained colocalized in the enlarged nuclear speckles, while SRSF2 gradually diffused into the nucleoplasm and no longer colocalized with PA^+^ (Fig. 1F and fig. S2, C and D), indicating that this PA^+^-enriched subcellular domain in oocytes is distinct from the SRSF2-labeled nuclear speckles. Therefore, we named this domain NPAD.

We also detected the localization of PA^+^ and PABPN1 in somatic cell lines (U2OS, HeLa, and mouse ovarian granulosa cells). In the nuclei of U2OS and HeLa cells, PABPN1 was unevenly distributed, whereas PA^+^ was uniformly distributed (fig. S2, E and F) and did not colocalize with the oocytes. In granulosa cells, PA^+^ condensates were detected in the nuclei, and PABPN1 colocalized with PA^+^ (fig. S2, E and F). Therefore, the formation of NPAD with poly(A) RNAs may be cell type specific. The RNA binding protein PABPN1 colocalized with PA^+^ and may be directly involved in the formation of the NPAD.

### NPAD formation depends on the transcription activity in oocytes and RNA binding ability of PABPN1

**Fig. 2.**
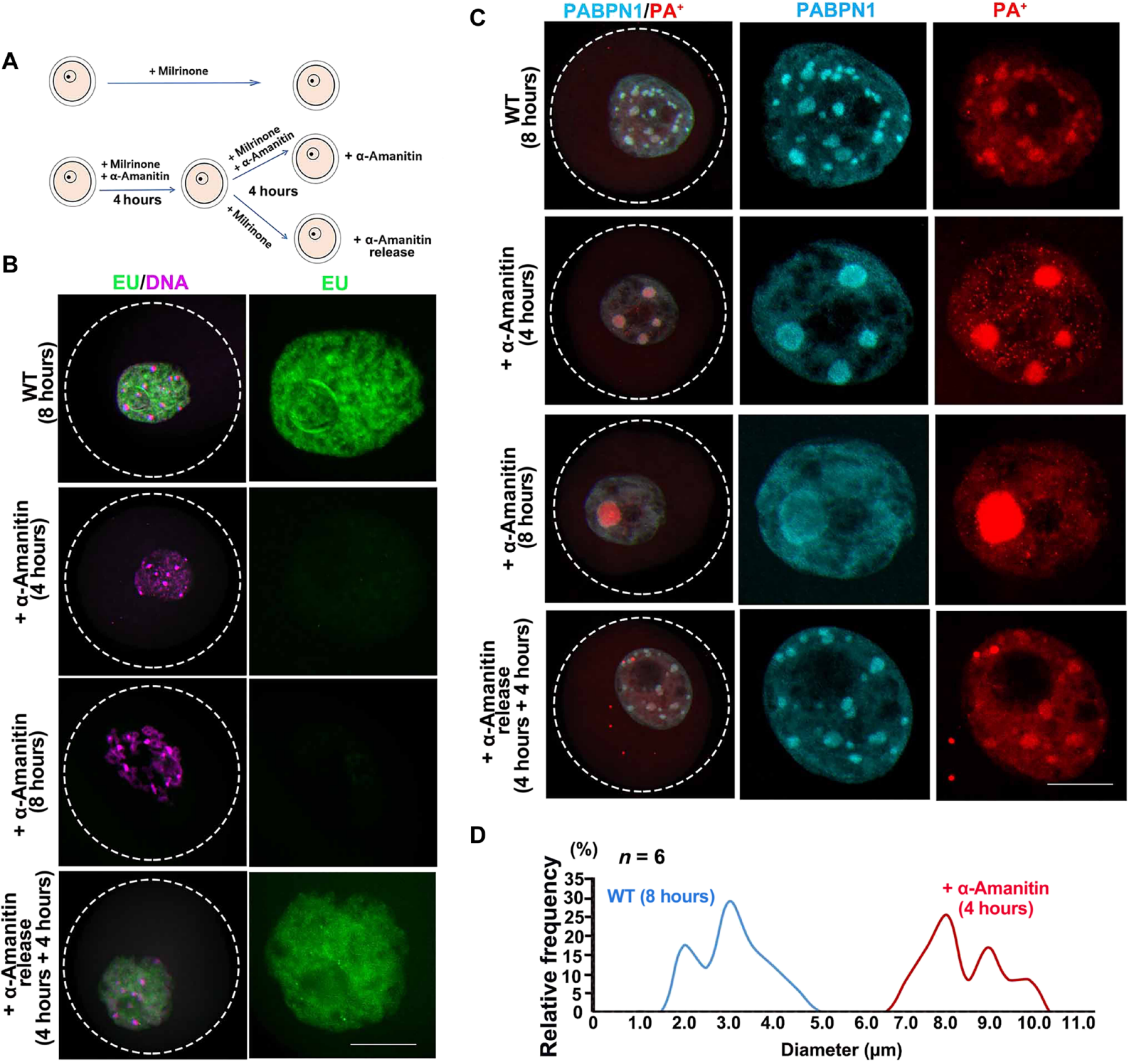
Inhibition of transcriptional activity alters the distribution of PABPN1 and PA^+^ in mouse growing oocytes. (**A**) Model diagram showing the differential processing of oocytes. The oocytes in the first group were retained as untreated wild type (WT); the second group was treated with α-amanitin for 8 hours, to inhibit transcriptional activity; the third group was treated with α-amanitin for 4 hours, to inhibit transcriptional activity, followed by its removal and incubation for another 4 hours. (**B**) EU incorporation to detect the transcription activity in growing oocytes. Scale bar, 10 μm. (**C**) Immunofluorescence and FISH results showed the localization of PABPN1 and PA^+^ in oocytes under different treatment conditions. Scale bar, 10 μm. (**D**) Statistical analysis of the size of PA^+^ in untreated WT and treated with α-amanitin for 4-hour oocytes.

NPAD is present in the nucleoplasm of growing oocytes that have active transcription but not in fully grown oocytes that are transcriptionally inactive. Therefore, we hypothesized that the formation of NPAD is directly related to transcription. When growing oocytes were cultured with the transcription inhibitor α-amanitin for 4 hours, ethynyl uridine (EU) staining showed that their transcriptional activity was inhibited (Fig. 2, A and B, and fig. S3A). When these oocytes were released into the medium without α-amanitin and cultured for another 4 hours, they regained their transcriptional activity (Fig. 2, A and B, and fig. S3A). Correspondingly, when the transcription in growing oocytes was inhibited, PABPN1 condensates aggregated and then diffused, while PA^+^ condensates fused into a few large dots (Fig. 2, C and D, and fig. S3B). In addition, when the oocytes resumed their transcriptional activity, the PABPN1 and PA^+^ condensates reformed (Fig. 2C). Therefore, the compartmentalized distribution of PABPN1 and NPAD in oocytes is directly related to their transcriptional activity.

**Fig. 3.**
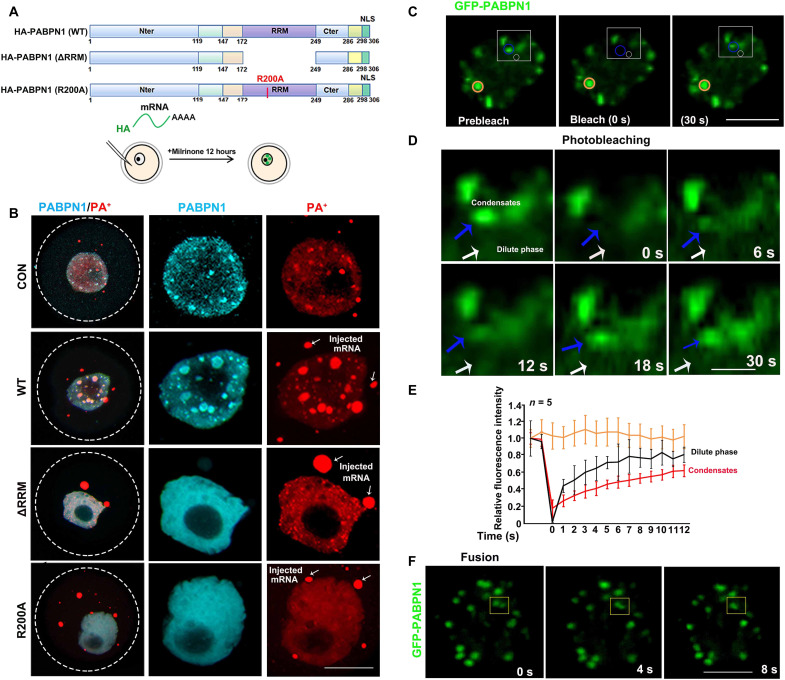
The RNA binding domain affects PABPN1 aggregation in the growing oocytes. (**A**) Schematic diagram of the different mutated forms of PABPN1. Full-length (WT) and deletion of RNA-recognized motif (ΔRRM) and mutated RNA-recognized motif (R200A). (**B**) Immunofluorescence results showing the localization of different mutant forms of PABPN1 (WT, ΔRRM, and R200A) and FISH results showing the PA^+^ distribution after overexpression of different mutant PABPN1s in growing oocytes. Scale bar, 10 μm. The white arrow indicates the injection of mRNA with poly(A) tails into the oocyte. (**C**) Image of GFP-PABPN1 before (left), during (middle), and after (right) photobleaching. Blue circles, PABPN1 condensates; white circles, PABPN1 diffused around. The yellow circles highlight the unbleached regions for comparison. The time relative to photobleaching (0 s) has been indicated. Scale bar, 10 μm. (**D**) Time lapse and view of condensate recovery for regions highlighted in blue and white in (C). The time relative to photobleaching has been indicated. Scale bar, 1 μm. Blue arrow, PABPN1 condensates; white arrow, PABPN1 diffused around. (**E**) Signal intensity relative to the prebleaching signal (*y* axis) versus time relative to photobleaching (*x* axis). Data have been presented as the average relative intensity ± SD (*n* = 5). The black, red, and yellow curves correspond to the signal changes in the different colored circles in (C). (**F**) Time-lapse images of growing oocyte nuclei expressing GFP-PABPN1. A condensate fusion event occurring in the growing oocyte nucleus, indicated using a yellow box in the figure.

The RRM of PABPN1, especially the R200 in RRM, is essential for the binding of PABPN1 with the poly(A) tails of mRNA (*6*). To investigate whether the formation of NPAD is affected by the interaction between PABPN1 and mRNA, we ectopically expressed hemagglutinin (HA)–tagged PABPN1 [wild type (WT), PABPN1-ΔRRM, and PABPN1-R200A] in growing oocytes by means of mRNA microinjection (Fig. 3A). The distribution of HA-PABPN1-WT in the growing oocytes was similar to that of endogenous PABPN1, showing compartmentalized distribution, but the condensates upon PABPN1 overexpression were larger than those formed by endogenous PABPN1 (Fig. 3B). PABPN1-ΔRRM and PABPN1-R200A were dispersed in the nucleoplasm (Fig. 3B). Overexpression of PABPN1-ΔRRM and PABPN1-R200A in growing oocytes disrupted the condensate distribution of PA^+^, which became larger in oocytes overexpressing PABPN1-WT but disappeared in oocytes overexpressing PABPN1-ΔRRM or PABPN1-R200A (Fig. 3B and fig. S3C). Therefore, the formation of NPAD is directly related to the binding ability of PABPN1 to mRNA. A few extra-large PA^+^ condensates were detected in the cytoplasm of oocytes overexpressing PABPN1 and its mutants (Fig. 3B). These were formed by the injected mRNAs that were polyadenylated in vitro.

We further explored whether PABPN1 exhibits the features of liquid-like condensates in vivo, characterized by internal dynamical reorganization and rapid exchange kinetics (*18*). When green fluorescent protein (GFP)–PABPN1 was overexpressed in growing oocytes by means of mRNA microinjection, it showed a condensate distribution in the germinal vesicles (Fig. 3C). Fluorescence recovery after photobleaching (FRAP) was used to study the dynamics of PABPN1 condensates in the growing oocytes. After photobleaching, PABPN1 condensates recovered their fluorescence on a time scale that was seconds slower than that of the dilute phase, suggesting condensate properties of PABPN1 and dynamic exchanges of PABPN1 between the condensates and dilute phase (Fig. 3, C to E). We also observed that the adjacent condensates fused, and these fusions exhibited liquid-like fusion properties (Fig. 3F). These results indicated that NPAD-containing PABPN1 have liquid-like properties in the oocyte nucleus.

### NPAD is formed by the condensation properties of PABPN1 and mRNA in the growing oocyte nucleus

The formation of membraneless NPAD by protein phase separation is an important way in which oocytes organize their nucleoplasm. In vitro phase separation assays with purified proteins are standard approaches to investigate proteins that form membraneless compartments. In recent years, various proteins have been purified and tested for their ability to undergo phase separation and form liquid condensates in vitro (*21*).

**Fig. 4.**
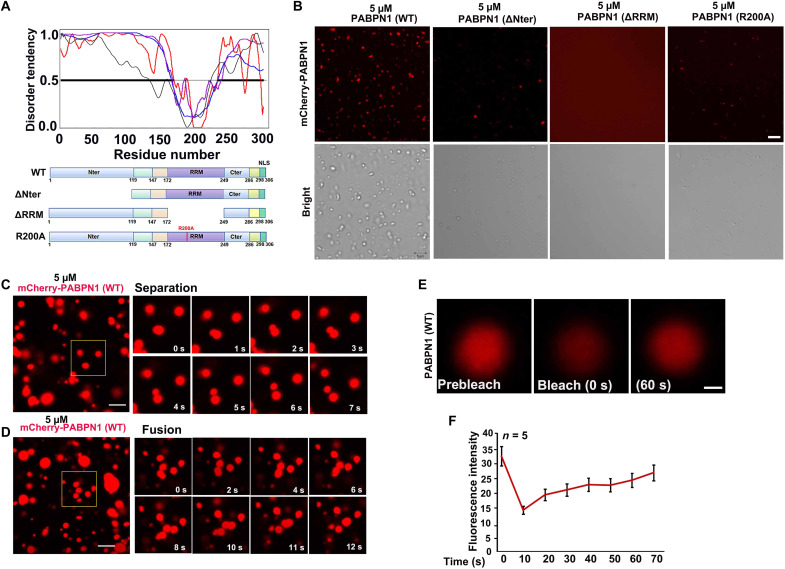
The purified PABPN1 undergoes condensation properties in vitro. (**A**) Disorder analysis of PABPN1 (306 amino acids). The plot shows the predicted disorder across full-length PABPN1 using various algorithms. The algorithms used were P-FIT (black line), VL3 (blue line), VL-XT (red line), and VSL2 (purple line). The diagram below shows the domains and boundaries of PABPN1 protein. Schematic diagram showing the four constructs of PABPN1: PABPN1 full length (WT), N-terminal deletion (ΔNter), RRM deletion (ΔRRM), and RRM mutation (R200A). (**B**) In vitro phase separation assay of mCherry-PABPN1 full length (WT), N-terminal deletion (ΔNter), RRM deletion (ΔRRM), and RRM mutation (R200A). Liquid condensates were formed after mixing 5 μM PABPN1 in the liquid system [20 mM tris-HCl (pH 7.5), 15 mM NaCl, 130 mM KCl, 5 mM KH_2_PO_4_, 1.5 mM MgCl_2_, bovine serum albumin (BSA) (1 mg/ml), and 10% polyethylene glycol, molecular weight 8000 (PEG-8000)]. Scale bar, 5 μm. (**C** and **D**) Time-lapse images of condensate separation and fusion events occurring in purified full-length PABPN1 in vitro. Scale bars, 10 μm. (**E**) Image of purified full-length PABPN1 before (left), during (middle), and after (right) photobleaching. The time relative to photobleaching (0 s) has been indicated. (**F**) Signal intensity (*y* axis) versus time relative to photobleaching (*x* axis). Data have been presented as the average relative intensity ± SD (*n* = 5).

Phase separation is a process in which a solution of proteins and other RNA and biomolecules spontaneously separates into two phases (*18*, *22*). Proteins that have the phase separation capacity often contain multiple self-interaction domains and have a high fraction of intrinsic disorder (*17*). We tested the disorder tendency of PABPN1 by IUPred (http://iupred.enzim.hu/) and identified an intrinsically disordered region in the N terminus of PABPN1 protein (Fig. 4A).

We tested whether purified PABPN1 can form liquid condensates in vitro. *Escherichia coli*–expressed and *E. coli*–purified mCherry-PABPN1 formed liquid-like condensates in vitro (Fig. 4B). The ability of PABPN1-ΔNter, PABPN1-ΔRRM, and PABPN1-R200A to undergo liquid-liquid condensation in vitro was notably weaker than that of full-length PABPN1 (WT), under similar conditions (Fig. 4B), indicating the importance of the intrinsically disordered region and RNA binding motif in liquid droplet formation. Compartments that form by phase separation can be very dynamic. They often have properties of liquid droplets and rapidly exchange components with their surroundings (*23*). Therefore, we tested whether the liquid droplets formed by purified PABPN1 can dynamically separate and fuse in vitro and exchange molecules with their surroundings. Images in Fig. 4 (C and D) showed that the PABPN1 condensates spontaneously separated and fused, which is characteristic for liquid-liquid phase separation. In addition, we assessed the mobility between PABPN1 condensates with bulk phases, by detecting the recovery of the fluorescent signal after photobleaching (Fig. 4E). Most fluorescence signals from the photobleached condensates could recover within 60 to 70 s (Fig. 4, E and F). These data demonstrated that PABPN1 dynamically exchanges molecules with the surrounding solution.

**Fig. 5.**
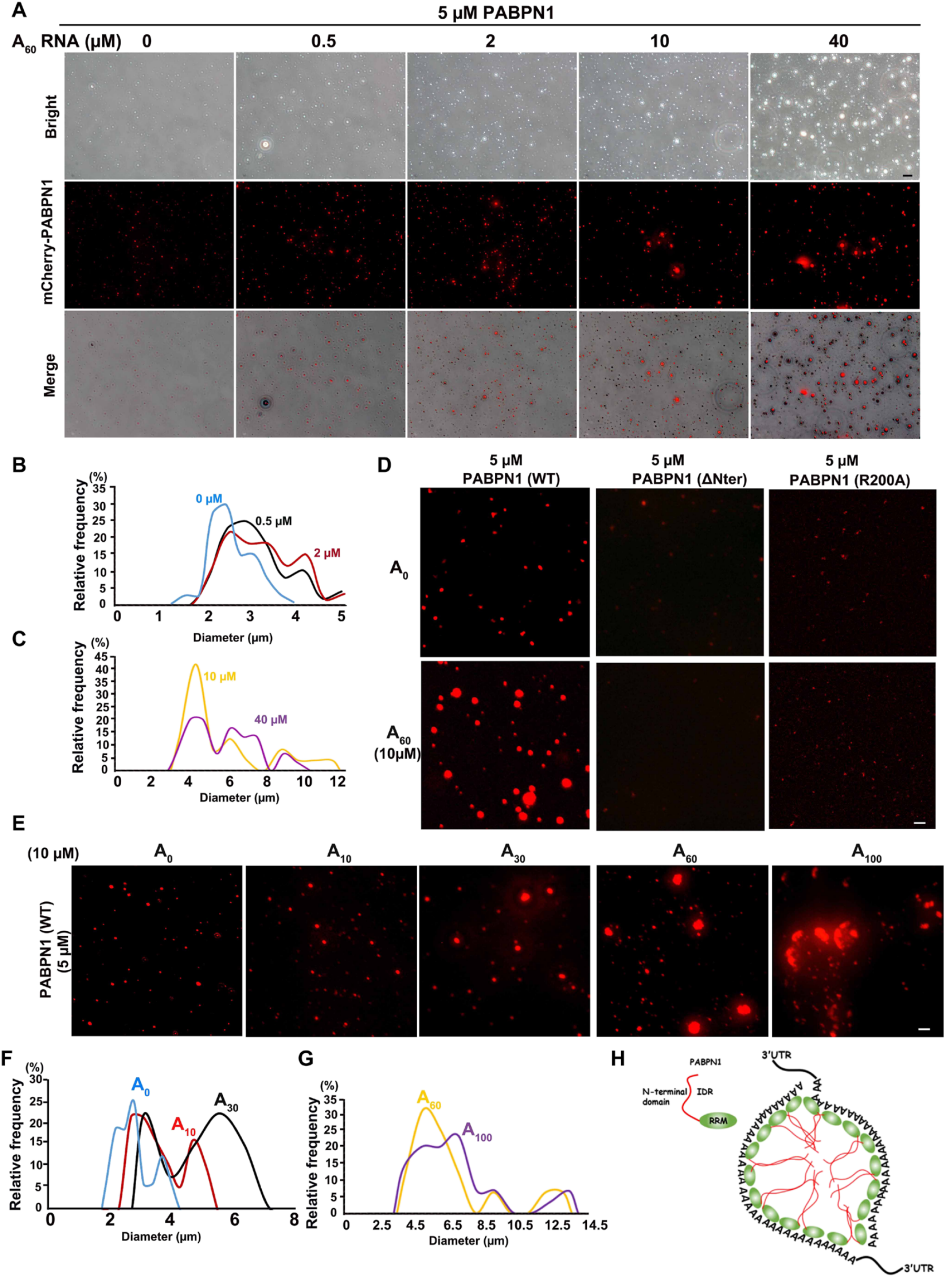
Addition of RNA containing a poly(A) tail promoted the condensation properties of PABPN1. (**A**) Representative images of condensation properties by mixing PABPN1 (5 μM) with different concentrations of A_60_ in physiological buffer [20 mM tris-HCl (pH 7.5), 15 mM NaCl, 130 mM KCl, 5 mM KH_2_PO_4_, 1.5 mM MgCl_2_, BSA (1 mg/ml), and 10% PEG-8000]. Scale bar, 10 μm. (**B** and **C**) Quantitative statistics of changes in the size of PABPN1 condensates with an increase in A_60_ concentration. (**D**) In vitro phase separation assay of PABPN1 (WT, ΔNter, and R200A) with or without 10 μM A_60_. Scale bar, 10 μm. (**E**) In vitro phase separation assay of PABPN1 with poly(A) tails of different lengths. Liquid condensates formed after mixing 5 μM PABPN1 (WT) in the liquid system (A) described previously with 10 μM poly(A) tails of different lengths. (**F** and **G**) Quantitative statistics of the size of PABPN1 condensates in (E). (**H**) The model shows that PABPN1-mRNA can promote the formation of NPAD by means of its condensation properties. IDR, intrinsically disordered region.

PABPN1 stimulates synthesis of the poly(A) tails of pre-mRNAs and minimally recognizes 10 to 14 adenine bases throughout its binding site (*24*–*26*). When poly(A) RNAs (A_60_) were added to the PABPN1 solution, PABPN1 colocalized with fluorescein isothiocyanate–A_60_ in vitro (fig. S4A). The sizes of PABPN1 condensates became larger as the concentration of A_60_ increased (Fig. 5, A to C), but the random RNA did not affect PABPN1 condensates (fig. S4, B and C). However, PABPN1-ΔNter and PABPN1-R200A did not form big condensates in vitro after addition of A_60_ (Fig. 5D). In addition, we added 10 μM poly(A)s of different lengths (A_10_, A_30_, A_60_, and A_100_) to 5 μM PABPN1 (Fig. 5E) and found that the longer the polymerized A RNA, the larger the condensate aggregation of PABPN1 (Fig. 5, E to G).

Together, these data suggested that both the Nter and RRM of PABPN1 are necessary for NPAD condensate formation (Fig. 5H). The longer the poly(A) tails of mRNA, the larger the NPAD organized by PABPN1, which is consistent with the gradual increase of NPAD in the growing oocytes under physiological conditions.

### *Pabpn1*-null oocytes cannot form NPAD normally

**Fig. 6.**
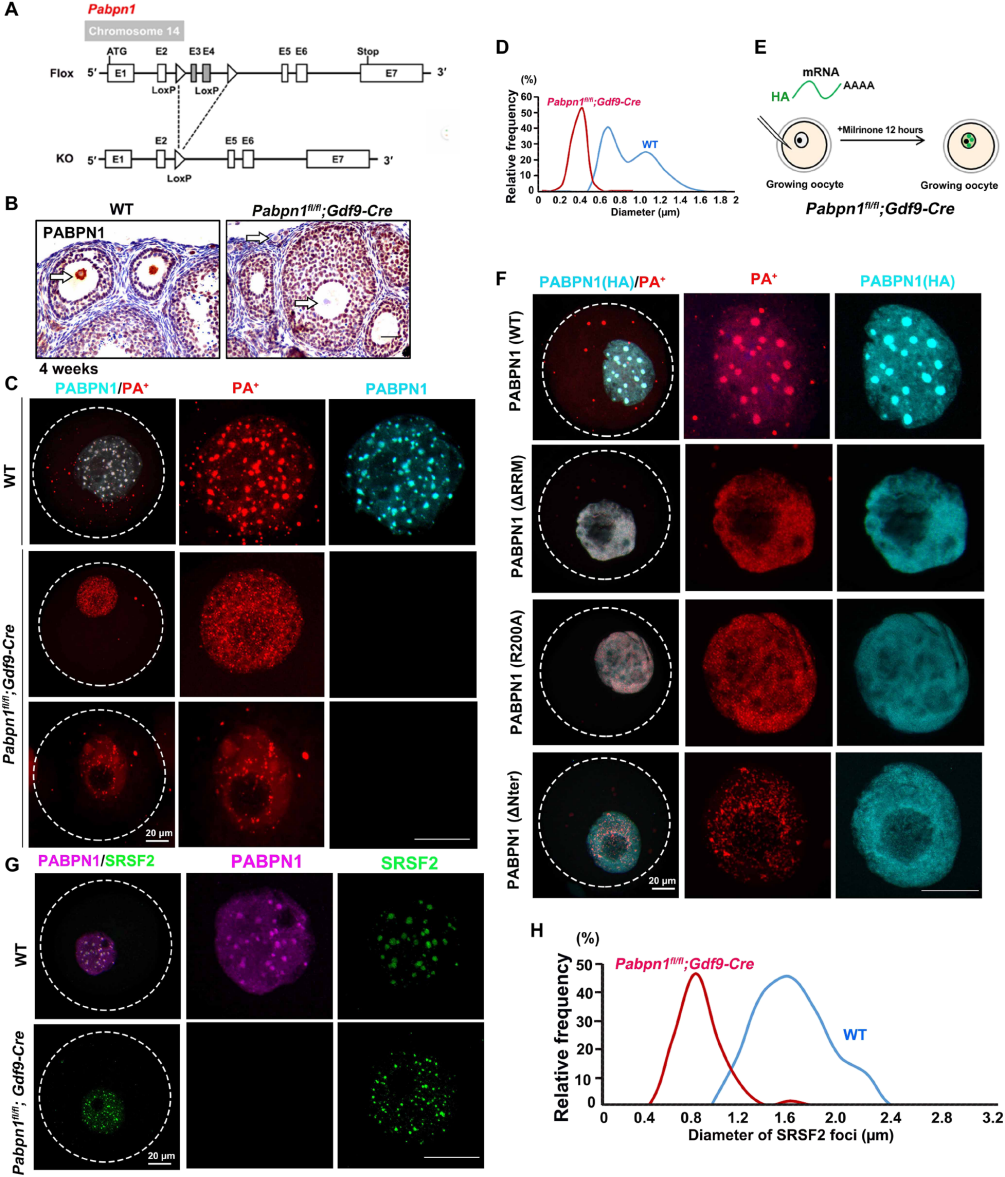
NPAD could not form normally in *Pabpn1*-null oocytes. (**A**) Diagram showing the strategy of *Pabpn1* conditional knockout. (**B**) Immunohistochemistry results showed that *Pabpn1* was specifically knocked out in oocytes of *Pabpn1^fl/fl^;Gdf9-Cre* mice. The arrow indicates the expression of PABPN1 in the oocyte nucleus. Scale bars, 20 μm. (**C**) Immunofluorescence and FISH results showing the distribution of PABPN1 and PA^+^ in WT and *Pabpn1-*deletion oocytes. (**D**) Statistical analysis of the size of PA^+^ in WT and *Pabpn1*-null oocytes. (**E**) Schematic diagram showing the injection of different forms of PABPN1 (WT, ΔRRM, R200A, and ΔNter) into the oocytes of *Pabpn1^fl/fl^;Gdf9-Cre* mice. (**F**) FISH and immunofluorescence results showing the localization of overexpressed PABPN1 (WT, ΔRRM, R200A, and ΔNter) and PA^+^ in *Pabpn1*-null oocytes. Scale bars, 20 μm. (**G**) Immunofluorescence results showing the distribution of PABPN1 and SRSF2 in WT and *Pabpn1-*deletion oocytes. (**H**) Statistical analysis of the size of SRSF2 foci in WT and *Pabpn1*-null oocytes.

To further verify that PABPN1 is necessary for the formation of NPAD in the oocyte nucleus and to determine its physiological function in vivo, we constructed *Pabpn1-*floxed mice and crossed them with the *Gdf9-Cre* transgenic mice, therefore specifically knocking out *Pabpn1* in oocytes as early as in the primordial follicle stage (Fig. 6A). The results of immunohistochemistry showed that PABPN1 was not detected in the oocytes of *Pabpn1^fl/fl^;Gdf9-Cre* mice, while it was detected in those of WT mice (Fig. 6B).

PA^+^ could not aggregate into large condensates in *Pabpn1*-null oocytes and was scattered in the form of small foci (Fig. 6, C and D, and fig. S3D). When we microinjected mRNAs encoding different forms of PABPN1 (WT, ΔRRM, R200A, and ΔNter) into *Pabpn1*-null growing oocytes and detected the formation of NPAD (Fig. 6E), FISH results showed that NPAD reformed in *Pabpn1*-null oocytes expressing exogenous PABPN1-WT but not in *Pabpn1*-null oocytes expressing PABPN1 mutants (ΔRRM, R200A, or ΔNter) (Fig. 6F and fig. S3E). SRSF2 also could not aggregate into large condensates in *Pabpn1*-null oocytes and was scattered in the form of small foci (Fig. 6, G and H, and fig. S3F). Collectively, these results indicate that PABPN1 is required for NPAD formation in growing mouse oocytes.

### *Pabpn1*-deletion in mouse oocytes causes female infertility and premature ovarian failure

**Fig. 7.**
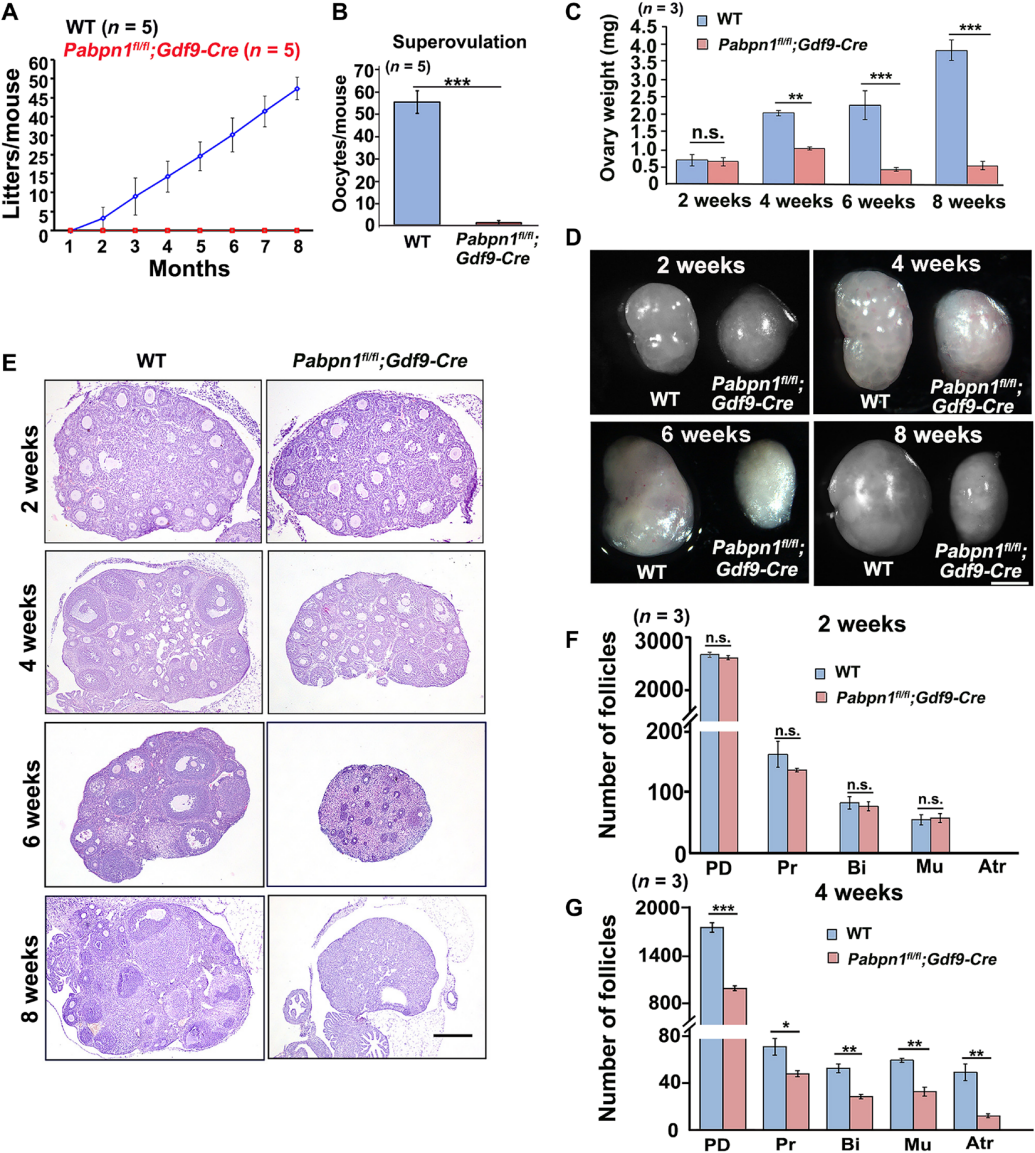
Phenotype analyses of *Pabpn1^fl/fl^;Gdf9-Cre* mice. (**A**) Fertility testing of WT and *Pabpn1^fl/fl^;Gdf9-Cre* female mice. *n* = 5 females for each genotype. (**B**) Number of oocytes that were ovulated by 4-week-old WT and *Pabpn1^fl/fl^;Gdf9-Cre* mice at 16 hours after human chorionic gonadotropin (hCG) injection. Five female mice of each genotype (*n*) were used in this experiment. Data have been represented as means ± SEM (***P < 0.001). (**C** and **D**) Representative ovarian weights (C) and images (D) of WT and *Pabpn1^fl/fl^;Gdf9-Cre* female mice at the indicated ages. Data have been represented as means ± SEM (**P < 0.01 and ***P < 0.001). n.s., nonsignificant. *n*, number of mice analyzed for each genotype. Scale bar, 500 μm. (**E**) H&E staining results showing ovarian histology of WT and *Pabpn1^fl/fl^;Gdf9-Cre* mice at the indicated ages. Scale bar, 100 μm. (**F** and **G**) Average number of follicles in ovaries of WT and *Pabpn1^fl/fl^;Gdf9-Cre* female mice at 2 and 4 weeks. Data have been represented as means ± SEM (*P < 0.05, **P < 0.01, and ***P < 0.001). PD, primordial follicles; Pr, primary follicles; Bi, secondary follicles with two layers of granulosa cells; Mu, secondary follicles with multiple layers of granulosa cells; Atr, antral follicles. *n* = 3 females for each genotype.

In the 8-month fertility tests, the *Pabpn1^fl/fl^;Gdf9-Cre* mice were found to be completely sterile (Fig. 7A). The 4-week-old WT mice ovulated approximately 55 mature oocytes after superovulation treatment, while almost no oocytes were ovulated by *Pabpn1^fl/fl^;Gdf9-Cre* mice of the same age (Fig. 7B).

The ovaries of 2-week-old WT and *Pabpn1^fl/fl^;Gdf9-Cre* mice were morphologically similar (Fig. 7, C and D). However, the ovaries of 4-, 6-, and 8-week-old *Pabpn1^fl/fl^;Gdf9-Cre* mice were smaller than those of WT mice (Fig. 7, C and D). Hematoxylin and eosin (H&E) staining showed that the *Pabpn1^fl/fl^;Gdf9-Cre* female mice underwent premature ovarian failure as early as at 6 to 8 weeks of age, characterized by oocyte depletion in the ovaries (Fig. 7, E to G). Follicle counting results showed that the number of follicles at each stage was normal in the ovaries of 2-week-old *Pabpn1^fl/fl^;Gdf9-Cre* mice (Fig. 7F) but significantly lower in mice that were 4 weeks of age (Fig. 7G).

Mouse vasa homolog (MVH) is a marker of germ cells. Immunohistochemistry showed that the number of germ cells in the ovaries of 4-week-old *Pabpn1^fl/fl^;Gdf9-Cre* mice was significantly lesser than that in WT mice. MVH-positive germ cells were completely depleted in the ovaries of 8-week-old *Pabpn1^fl/fl^;Gdf9-Cre* mice (fig. S5, A and B). We detected the cell apoptosis on ovarian sections by immunohistochemistry of cleaved caspase 3, which is a widely recognized marker of apoptosis. The results showed that there were more apoptosis signals in granulosa cells on the 4-week-old *Pabpn1^fl/fl^;Gdf9-Cre* ovarian sections than in the WT ovarian sections, suggesting that *Pabpn1* deletion in oocytes caused the apoptosis of the surrounding granulosa cells and follicle atresia (fig. S5, C and D).

### Knockout of *Pabpn1* affects the transcriptome in oocytes

Our study proved that the formation of NPAD is related to transcription. For this, we collected WT and *Pabpn1*-knockout oocytes for transcriptome sequencing and analyzed them in three sets; the samples had similar global complementary DNA (cDNA) length distributions (fig. S6, A and B) and showed high correlations (fig. S6C).

**Fig. 8.**
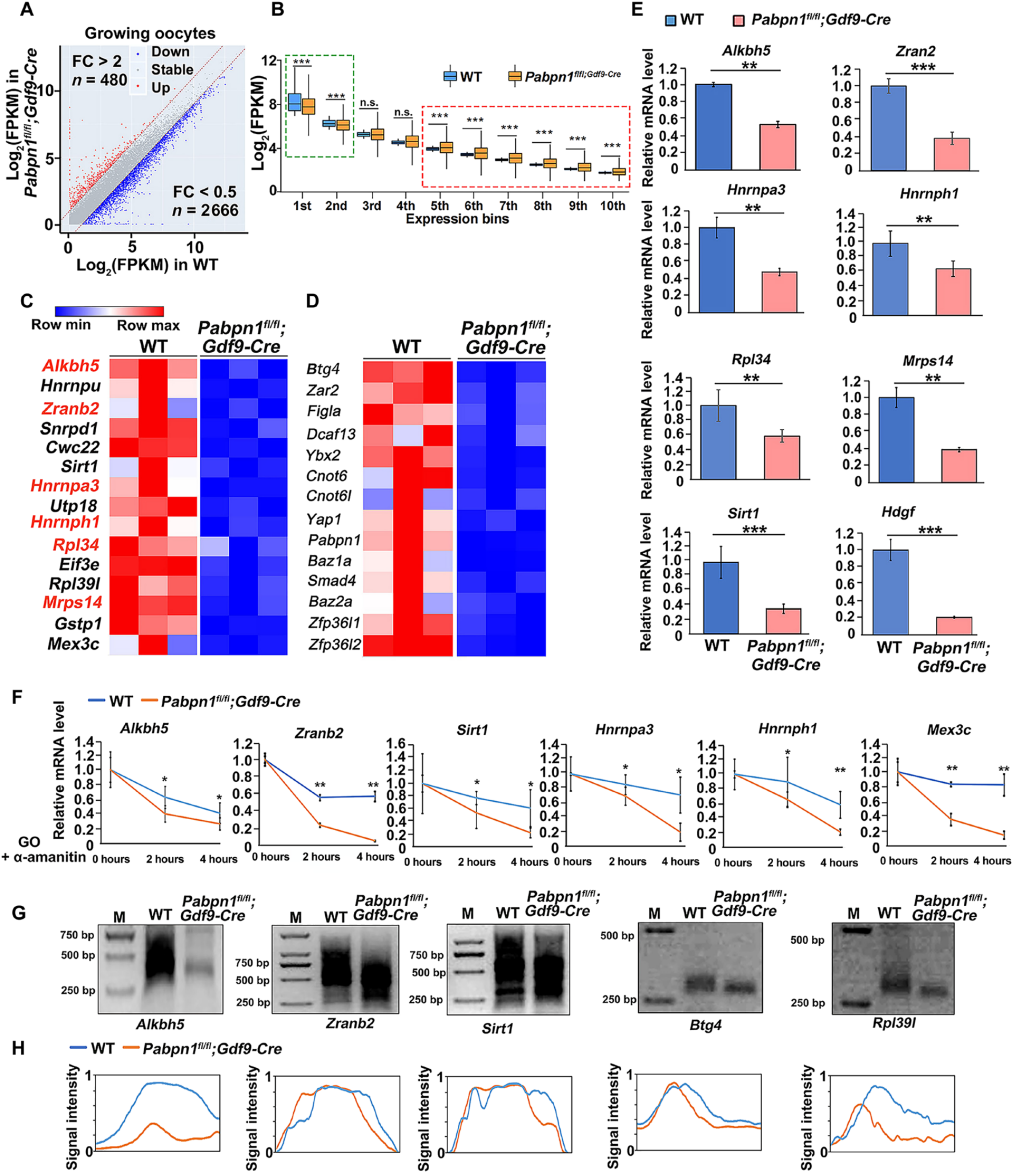
Transcriptomic analyses of *Pabpn1*-null oocytes. (**A**) Scatter plot of the relative transcript levels in WT and *Pabpn1*-deletion growing oocytes. Transcripts (*n*) whose levels decreased or increased by more than twofold have been highlighted in blue and red, respectively. FC, fold change. (**B**) Box plot showing gene expression levels of WT and *Pabpn1^fl/fl^;Gdf9-Cre* mouse oocytes at the growing stage. Genes were divided into 10 bins according to their expression levels in WT oocytes. The box indicates the upper and lower quantiles, the thick line in the box indicates the median, and the whiskers represent the 2.5th and 97.5th percentiles. ***P < 0.001, as assessed using two-tailed Student’s *t* test. Green box, transcripts with high levels in growing oocytes; red box, transcripts with low levels in growing oocytes. (**C** and **D**) Heatmap of the transcripts related to mRNA metabolism (C) and oocyte development (D) that showed decreased levels in *Pabpn1^fl/fl^;Gdf9-Cre* mice, as compared to those in WT mice. (**E**) Quantitative RT-PCR results showing the relative levels of the indicated transcripts in WT and *Pabpn1*-deletion oocytes after transcription inhibition by α-amanitin. Error bars represent the SEM. Statistical analysis was performed using Student’s *t* test. **P < 0.01 and ***P < 0.001. (**F**) RT-qPCR results showing the changes in transcript levels in WT and *Pabpn1*-null oocytes. Data have been represented as means ± SEM from at least three independent experiments and have been compared using one-way analysis of variance (ANOVA). *P < 0.05 and **P < 0.01. (**G**) Changes in poly(A) tail length of transcripts from WT and *Pabpn1^fl/fl^;Gdf9-Cre* mouse oocytes, as assessed using poly(A)-tail assay. (**H**) Relative signal intensity (*y* axis) and length of PCR products based on mobility (*x* axis) in (G). cKO, conditional knockout.

We calculated gene expression levels of transcripts in terms of fragments per kilobase per million mapped reads (FPKM) and evaluated the relative mRNA copy number using the External RNA Controls Consortium (ERCC) RNA spike-in. The overall transcript level of *Pabpn1*-knockout oocytes was not significantly different from that of WT oocytes (fig. S6D). Numerous transcripts were increased (480) or decreased (2666) in *Pabpn1*-null oocytes, as compared to those in WT oocytes (Fig. 8A). Transcripts with higher expression levels (indicated using a green box) in WT growing oocytes decreased significantly after deletion of *Pabpn1*, whereas transcripts with lower expression levels (indicated using a red box) in WT growing oocytes increased after deletion of *Pabpn1* (Fig. 8B). The transcript level of *Pabpn1* in *Pabpn1*-null oocytes was significantly lower than that in WT oocytes, indicating that knockout of *Pabpn1* was effective (fig. S6E). The transcript level of *Pabpn1l* (homolog of *Pabpn1*) was similar in *Pabpn1*-null and WT oocytes (fig. S6F).

Gene ontology (GO) analysis of the increased transcripts was enriched in the terms of phospholipid biosynthetic process, protein glycosylation, and nucleic acid binding (fig. S6G). Many of the down-regulated transcripts in *Pabpn1*-null oocytes were encoded by genes related to translation, mRNA processing, RNA splicing, and RNA binding (fig. S6, H and I, and Fig. 8C). Transcripts related to oocyte development also decreased in *Pabpn1*-null oocytes (fig. S6J and Fig. 8D).

We further used reverse transcription quantitative polymerase chain reaction (RT-qPCR) to detect transcript levels in WT and *Pabpn1*-null oocytes. Consistent with the transcriptome sequencing results, levels of many transcript related to RNA processing and translation were decreased in *Pabpn1*-knockout oocytes (Fig. 8E). To exclude the effect of transcription activity on the mRNA levels in growing oocytes, 50 μM α-amanitin was used to repress de novo gene transcription in cultured WT and *Pabpn1*-deleted oocytes. The mRNA levels of the indicated transcripts gradually decreased in WT oocytes with time (Fig. 8F), whereas the decay of these transcripts was significantly accelerated in *Pabpn1*-null oocytes (Fig. 8F). In eukaryotes, the poly(A) tail is a major determinant of the steady state of mRNAs. Therefore, we tested the poly(A) tail of specific transcripts in WT and *Pabpn1*-deleted oocytes using a poly(A)-tail assay. The poly(A) tails of these transcripts were remarkably shortened in *Pabpn1-*deletion oocytes, as compared to those in WT oocytes (Fig. 8, G and H). These findings revealed that the normal formation of NPAD is essential for the stability of transcripts including those related to mRNA metabolism during oocyte growth.

### *Pabpn1* knockout results in shortened 3′UTR isoform transcripts

**Fig. 9.**
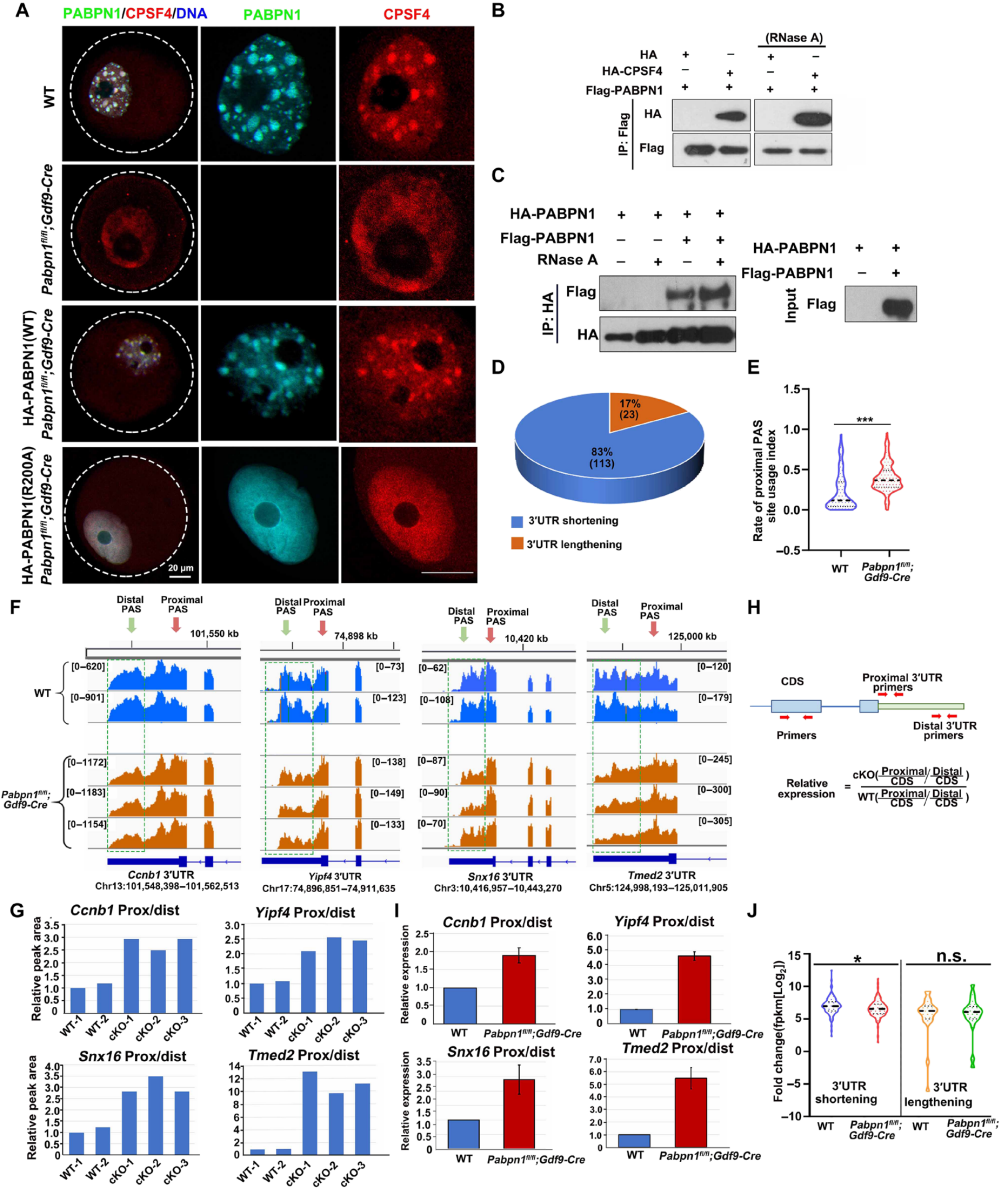
Transcript 3′UTR selection in *Pabpn1*-knockout oocytes. (**A**) Immunofluorescence results showing the distribution of PABPN1 and CPSF4 in WT and *Pabpn1^fl/fl^;Gdf9-Cre* mouse oocytes. Scale bar, 20 μm. (**B** and **C**) Co-IP experiment showing the interaction of PABPN1 with CPSF4 and PABPN1 (C). (**D**) Number of transcripts with 3′UTR shortening or lengthening in *Pabpn1^fl/fl^;Gdf9-Cre* mice oocytes, as compared to those in control oocytes. (**E**) Comparison between the proximal PUI calculated in WT and *Pabpn1^fl/fl^;Gdf9-Cre* mouse oocytes. The analysis included 136 transcripts that showed a significant shift in the PAS usage. (**F**) Examples of transcripts showing significant 3′UTR shortening, manifested by prominent decrease peaks near the distal CS relative to the proximal ones in *Pabpn1*-deletion oocytes, as compared to those in WT samples. Transcripts with green boxes represent the distal CS levels. Green arrow, distal PAS; red arrow, proximal PAS. (**G**) The ratios of the proximal to distal peak area are shown in (F), normalized to the average of the two controls. (**H**) The primer design pattern diagram detects the proportion of long and short isomers in the transcript. Separately designed primer sequences to detect the CDS, proximal, and distal isoforms of a specific transcript. The proportions of the proximal and distal isomers of the same transcript were normalized to the CDS. (**I**) To confirm the results of the 3′UTR selection, the same samples as in (F) were subjected to qRT-PCR. (**J**) RNA sequencing results showing the relative levels of transcripts that displayed 3′UTR shortening or lengthening in WT and *Pabpn1^fl/fl^;Gdf9-Cre* mice oocytes. Error bars represent the SEM. *P < 0.05 by as assessed using the two-tailed Student’s *t* test.

The newly transcribed mRNAs are transported out of the nucleus after processing in the nucleus, and CPSF4 is an important subunit of the splicing complex in the nucleus (*27*). Immunofluorescence results showed that CPSF4 and PABPN1 displayed condensates that colocalized in the nuclei of the WT growing oocytes (Fig. 9A). The condensate distribution of CPSF4 was lost in *Pabpn1*-knockout oocytes, and they diffused in the nucleus (Fig. 9A). When PABPN1-WT was overexpressed in *Pabpn1*-null oocytes, CPSF4 reverted to condensate distribution, whereas when the mRNA binding mutation PABPN1-R200A was supplemented, CPSF4 could not restore the formation of the condensates (Fig. 9A). The above results showed that PABPN1 promoted the condensate distribution of CPSF4.

Protein complex immunoprecipitation (co-IP) results showed that CPSF4 and PABPN1 interacted independently of mRNA (Fig. 9B). In addition, PABPN1 itself also had RNA-independent interactions (Fig. 9C). Therefore, PABPN1 performs at least two functions under physiological conditions: PABPN1 can (i) promote the formation of NPAD and (ii) recruit mRNA splicing–related factors, to promote the processing of newly transcribed mRNA.

Only a few transcripts listed in fig. S7A showed abnormal intron splicing in *Pabpn1-*deleted oocytes, as compared to those in WT oocytes (fig. S7, A and B). In addition to intron splicing, mRNA processing in the oocyte nucleus also includes 3′UTR splicing, which is a two-step process consisting of endonucleolytic cleavage and addition of a poly(A) tail (*28*). An upstream hexamer (AAUAAA or AUUAAA) PAS and a downstream UG-rich sequence that recruits CPSF, both of which are required for the processing (*29*, *30*).

We then examined the effect of *Pabpn1* deletion on PAS selection. We identified 136 transcripts that showed a significant shift in cleavage site (CS) usage in *Pabpn1*-deletion samples versus control samples (Fig. 9D) (*P* < 0.05; table S7). GO analysis of the significant shift in CS usage transcripts was related to translation, RNA transport, and cell cycle (fig. S7C). In more than 80% of these transcripts, the shift in CS usage was toward the proximal PASs (*P* < 0.05; table S7), indicating an extensive 3′UTR shortening in the absence of PABPN1 (Fig. 9D). We defined a PAS usage index (PUI; table S7) to quantify the relative usage of each PAS in a transcript. Comparison of the proximal PUI distribution revealed a significant shift in CS usage transcripts in *Pabpn1*-deletion samples versus control samples, which further demonstrated the notably enhanced usage of proximal PASs in the absence of PABPN1 (Fig. 9E). We identified some transcripts (*Ccnb1*, *Yipf4*, *Snx16*, and *Tmed2*) that had 3′UTR shortening in the absence of PABPN1 and shorter 3′UTR isoforms (Fig. 9, F and G). We further validated the increase in the level of the short isoform relative to the long isoform for selected genes using RT-qPCR (Fig. 9, H and I). These results strongly suggested that *Pabpn1* knockout in oocytes leads to a significant shift of usage toward proximal CSs, which causes more shortening of the 3′UTR isoforms in the transcripts.

At the molecular level, the mRNA in NPAD is spliced at different CSs on the 3′UTR, to produce different isomeric forms. The levels of transcripts showing significant 3′UTR shortening were lower in *Pabpn1*-null oocytes than in WT oocytes, whereas the levels of transcripts showing significant 3′UTR lengthening were not significantly different between the two groups of oocytes (Fig. 9J). Therefore, PABPN1-dependent selection of CSs on the 3′UTR of transcripts during oocyte growth is crucial for transcript level and female fertility.

### PABPN1L is not involved in the regulation of NPAD in oocytes

To test whether PABPN1L is expressed in growing oocytes and has a physiological function that overlap with that of PABPN1 during this process, we detected the distribution of PABPN1L in growing oocytes by immunofluorescence. The PABPN1L was dispersed in the nucleus of oocytes and did not colocalize with SRSF2 to form condensates, which is different from the localization of PABPN1 (fig. S8A). The distribution of PABPN1L indicated that it was not involved in the formation NPAD in oocytes. In addition, *Pabpn1l* deletion did not affect the formation NPAD in oocytes (fig. S8B). Overexpressing of PABPN1L could not rescue the formation failure of NPAD in *Pabpn1*-null oocytes (fig. S8C). The structural differences between PABPN1 and PABPN1L determine that PABPN1L may not be involved in NPAD formation in the oocyte nucleus, because of the lack of the intrinsically disordered region in the N terminus of PABPN1L (fig. S8D). Furthermore, overexpressing PABPN1L in *Pabpn1*-knockout oocytes cannot rescue the gene transcription abnormalities caused by PABPN1 deletion during oocyte maturation (fig. S8E). This is consistent with the observation that PABPN1L does not play a role in the formation of NPAD. The above results indicate that PABPN1L does not have a redundant role with PABPN1 in the formation of NPAD during oocyte maturation.

## DISCUSSION

Mammalian oocytes, the largest cells in the body, are precisely regulated during their growth and development processes. These have no transcriptional activity during ovulation and fertilization and rely on the translation of maternal mRNA originally stored in the oocytes to regulate cell activities (*31*–*34*). The growing oocytes carry out active transcription activity and store a large number of maternal factors for the subsequent MZT process (*35*–*37*). After fertilization, the genome of the embryo is still in a state of transcriptional inhibition, and the embryo’s own genome does not start full transcriptional activation until the two-cell stage (*38*). Therefore, normal storage of maternal factors in growing oocytes is essential for the subsequent development of oocytes.

There are many different domains and nucleosomes in the cell nucleus that are involved in the different aspects of gene expression (*20*, *39*). This compartmentalization in the nucleus is more efficient in conducting various life activities (*40*). The distribution of PABPN1 is not uniform but has a similar location to that of SRSF2 (nuclear speckles, a subcellular membraneless structure) in the nucleus, which is rich in transcription, RNA processing, and export-related factors, as well as a variety of noncoding RNAs. The SRSF2 domain is the hub of transcription, mRNA processing, and export. It promotes the expression of related genes by increasing the concentration and dynamic exchange of factors related to mRNA metabolism (*20*).

In this study, we found that PABPN1 can promote mRNA with poly(A) tails to form NPAD structure that is different from SRSF2 in growing oocytes. Moreover, the distribution of NPAD gradually diffused with the development and maturity of the oocytes. The nuclear compartmentalization distribution of NPAD was found to be directly related to transcriptional activity and the developmental stage of oocytes. This conclusion is supported by the following experimental evidence. (i) Under physiological conditions, PABPN1 and poly(A)-tailed mRNAs were colocalized in the growing oocyte, but they gradually dispersed as the oocyte matured; (ii) SRSF2 was not completely colocalized with PABPN1 and PA^+^ (Fig. 1F); (iii) after inhibition of transcription, PABPN1 and NPAD lost their condensate distribution in the growing oocytes (Fig. 2C); and (iv) in growing oocytes, the condensate positions of PABPN1 and NPAD were directly related to its RNA binding capability. These results indicated that NPAD is directly related to the binding of PABPN1 to newly transcribed mRNA.

Recent studies have shown that macromolecules and membraneless organelles perform biological functions such as innate immune signaling, gene transcription, assembly of spindle apparatus, and heterochromatin formation by means of condensation (*41*–*45*). In mouse oocytes (which are different from somatic cells that have their own unique properties), there is still limited information regarding whether condensation properties affect reproductive activities.

In the current study, we hypothesized that PABPN1 forms NPAD by virtue of its condensation properties in growing mouse oocytes and is essential for female reproduction. The Nter of PABPN1 contains a low-complexity sequence domain followed by an RRM (Fig. 4A). This structure is similar to the previously reported structure of hnRNPA1, which is an RBP participating in the formation of stress granules, owing to its liquid-liquid condensation properties (*46*, *47*). The following evidence supports that PABPN1-mRNA promotes the normal formation of NPAD through condensation: (i) The purified PABPN1 can form a condensate morphology in vitro, similar to that in vivo (Figs. 3 and 4). (ii) Purified PABPN1 can fuse and exchange materials frequently with the outside conditions, both in vitro and in vivo (Figs. 3 and 4). (iii) The Nter and RRM of PABPN1 are essential for the formation of NPAD through condensation (Figs. 3 and 4). On the other hand, the size of NPAD is directly related to the length of the poly(A) tail of mRNAs (Fig. 5).

**Fig. 10.**
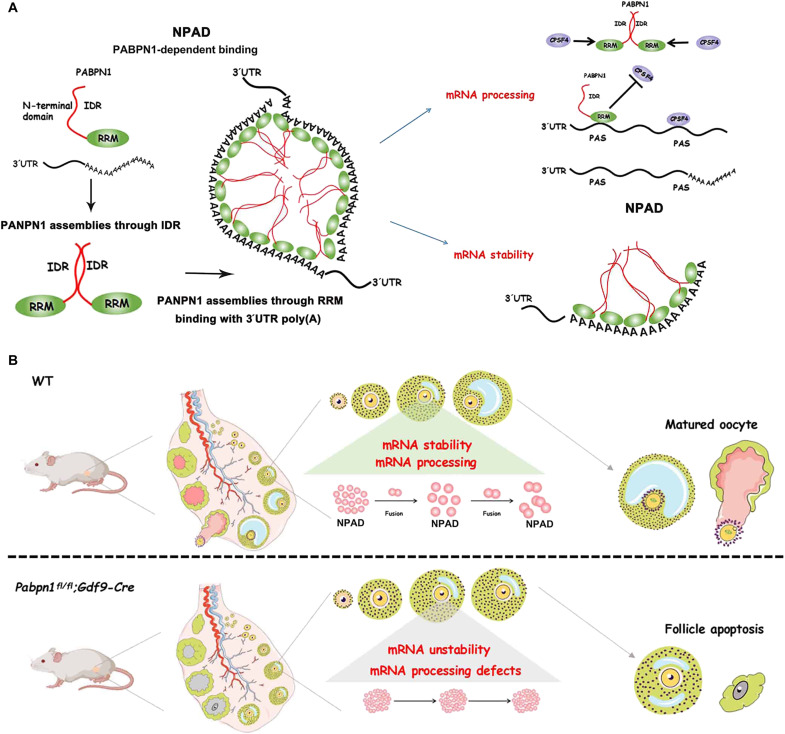
Schematic diagram showing the formation and physiological functions of NPAD in growing oocytes, as promoted by the condensation properties of PABPN1. PABPN1 promotes the formation and fusion of NPAD through condensation in growing oocytes. At the molecular level, NPAD promotes the formation of long 3′UTR transcripts by binding with proximal PAS (thus preventing the binding of the splicing factor CPSF4) and maintains the stability of mRNA by binding with the poly(A) tails of transcripts. At the physiological level, the normal formation of NPAD is essential for oocyte development, follicle growth, and ultimately female reproduction.

In addition, previously unidentified evidence, including the fact that the oocytes from *Pabpn1^fl/fl^;Gdf9-Cre* mice could not form NPAD, proves that PABPN1 is essential for the formation of NPAD under physiological conditions (Fig. 6). The knockout of *Pabpn1* affected the condensate localization of the splicing factor CPSF4. Our study proved that NPAD is the hub of mRNA processing, and PABPN1 further recruits other splicing factors to promote the storage and processing of mRNA in growing oocytes (Fig. 10A). In addition, PA^+^ condensates could serve as biomarkers for RNA processing dysfunction in the development of oocytes. This study is likely to offer great mechanistic and potentially therapeutic insights into these diseases.

There are still many questions surrounding the role of NPAD that have been left unanswered by this study. The condensate distribution of NPAD is directly related to its transcriptional activity. However, more evidence is needed to unravel the mechanism of NPAD formation, thus warranting further investigation. In addition, it has been previously reported that PABPN1 is involved in mRNA transport from the nucleus to the cytoplasm (*8*). While *Pabpn1*-null oocytes have mRNA in the nucleus and cytoplasm, the absence of *Pabpn1* may not affect the transport of mRNA from the nucleus to the cytoplasm. Thus, further studies are needed to determine whether PABPN1 is directly involved in the transport of specific mRNAs (*48*, *49*).

Transcriptome sequencing results showed that the overall maternal mRNA level of *Pabpn1*-null oocytes was not significantly different from that of the WT oocytes. While *Pabpn1* may affect some specific transcripts, PABPN1 may directly affect the transcriptional activity.

It has been reported that PABPN1 is associated with RNA polymerase II during transcription (*50*). In addition to PABPN1, CPSF4, and mRNA, NPAD in the nucleus should possibly contain other components. With the developed of RNA and DNA SPRITE methods, RNA and DNA in the spatial organization of NPAD could be mapped (*51*). Further investigations are required to explore the components of this membraneless organelle and to reveal the reason why it is not completely colocalized with SRSF2.

The in vivo significance of NPAD in growing oocytes was demonstrated using *Pabpn1^fl/fl^;Gdf9-Cre* knockout mice. *Pabpn1^fl/fl^;Gdf9-Cre* knockout female mice were infertile and had severe primary ovarian insufficiency (POI) (Fig. 10B). The NPAD mediated by phase separation of PABPN1 provides a gathered compartment in the nucleus, promoting the formation of long transcript isoforms, which in turn affect the stability of transcripts and the translation level of the oocyte.

*Pabpn1*-null led to NPAD formation defects and thus affected the stability of transcripts involved in oocyte development, such as *Ybx2*, *Baz1a*, *Cnot6*, and *Cnot6l*. *Pabpn1*-null oocytes failed to develop to the fully grown stage and in turn resulted in POI. PABPN1 is a protein that can bind to the poly(A) tails of mRNA and is essential for transcript stability. *Pabpn1* knockout results in a decline in transcripts relating to mRNA processing, RNA splicing, translation, oocyte development, and female reproduction. Among these newly identified PABPN1 target transcripts, *Alkbh5* encodes a mammalian RNA demethylase that affects mRNA export and RNA metabolism as well as the assembly of mRNA processing factors in nuclear speckles. *Alkbh5*-deficient male mice are infertile and have increased m6A in mRNAs of meiotic metaphase-stage spermatocytes (*52*). *Hnrnph1* and *Hnrnpa3* functionally associate with RNA splicing. The levels of these two transcripts are drastically reduced in *pabpn1*-deficient oocytes, potentially affecting RNA foci and transcript splicing in oocytes (*53*, *54*). In addition, the translation-related transcript *Rpl34* is also a target gene of PABPN1. The decreases of *Rpl34* transcript level after *Pabpn1* deletion may cause a decrease in the overall translation level in the knockout oocytes, resulting in abnormal oocyte development (*55*).

The condensation and compartmentalization of PABPN1 is one of the mechanisms that lead to infertility of *Pabpn1*-knockout mice. Other mechanisms related to PABPN1’s own structure and function underlying POI remain to be further explored.

Together, our study revealed the presence of an NPAD that promotes processing of maternal factor mRNA in growing oocytes. We further confirmed that PABPN1-RNA promoted the formation of NPAD through its condensation properties. We then verified our hypothesis using *Pabpn1^fl/fl^;Gdf9-Cre* knockout mice and explored the physiological significance of NPAD. Last, we investigated the physiological activities that occur in NPAD at the molecular level.

## MATERIALS AND METHODS

### Animals

The *Pabpn1^fl/fl^* mouse strain was constructed using CRISPR-Cas9–based gene targeting. *Pabpn1l^−/−^* mice have been previously reported (*6*). All mice had a C57BL/6J genetic background. All animal experiments were conducted in accordance with the guidelines and regulations of Zhejiang University, and the experimental protocol (ZJU20210252) was approved by the Zhejiang University Institutional Animal Care and Research Committee.

### Preparation of mRNAs for microinjections

To prepare mRNA for microinjection, the expression vectors were linearized and in vitro transcribed using the SP6 message mMACHINE Kit (Invitrogen, AM1340). Poly(A) tails [~200 to 250 base pairs (bp)] were added to the transcribed mRNAs using the mMACHINE kit (Invitrogen, AM1350). The in vitro–transcribed mRNAs were resuspended in nuclease-free water by lithium chloride precipitation method.

### Microinjection of oocytes

Growing oocytes were incubated in M2 medium containing 2 μM milrinone to inhibit spontaneous germinal vesicle breakdown (GVBD) for later microinjection. All injections were performed using an Eppendorf transferman NK2 micromanipulator. Approximately 5 to 10 pl of RNAs at a concentration of 500 ng/μl were microinjected to each oocyte. After microinjection, oocytes were cultured in M16 medium containing 2 μM milrinone at 37°C and 5% CO_2_.

### Histological and immunohistochemistry

After dehydration, the ovaries of the mice were embedded in paraffin and sectioned. The sections were deparaffinized in xylene three times (for 5 min each), rehydrated in anhydrous ethanol and 95% ethanol twice (for 5 min each), and stained with H&E.

For immunohistochemical analyses, the sections were incubated in 3% H_2_O_2_ for 10 min, cooled down to room temperature after boiling in 10 mM sodium citrate buffer (pH 6.0) for 15 min, washed, blocked with 10% donkey serum for 60 min, and incubated with primary antibodies overnight at 4°C. The slides were then washed and incubated with biotin-labeled secondary antibodies. The antibodies were further detected using the VECTASTAIN ABC kit and 3,3′-diaminobenzidine peroxidase substrate kit (Vector Laboratories, Burlingame, CA, USA). The slides were then counterstained with H&E and imaged under an 80i microscope equipped with a camera (Nikon, Tokyo, Japan).

### Immunofluorescence

Oocytes were fixed in 4% paraformaldehyde (PFA) at room temperature for 30 min and then incubated in 0.3% Triton X-100 in phosphate-buffered saline for 30 min. Antibody staining was performed using standard protocols described previously (DOI: 10.1242/dev.144410) (*56*). The antibodies used are listed in table S1. Images of oocytes were detected using an LSM 710 confocal microscope (Zeiss, Jena, Germany). Semiquantitative analysis of fluorescence signals was performed using ImageJ (NIH, Bethesda, MD, USA).

### Western blot

Oocytes were lysed in loading buffer (containing β-mercaptoethanol) and heated at 95°C for 10 min. SDS–polyacrylamide gel electrophoresis and Western blot were performed according to standard procedures (DOI: 10.1002/advs.202003636) (*57*).

### FISH assay

Oocytes were washed in 0.2% bovine serum albumin (BSA) and transferred to 4% PFA for 25 min at room temperature. Next, the oocytes were permeabilized at room temperature for 20 min with 2% Triton X-100 and 1× RNA ribonuclease (RNase) inhibitor and transferred to a wash buffer (preheated to 37°C) containing 1× RNA RNase inhibitor for 15 min. Following that, the oocytes were transferred a hybridization buffer into which a probe was added and allowed to incubate in a thermostat incubator, overnight at 37°C in the dark. This was followed by washing of the oocytes in warm wash buffer for at least 50 min at 37°C and a rinse in 2× saline-sodium citrate for 2 min at room temperature (*58*). Next, the oocytes were incubated with appropriate primary and second antibodies, similar to those used in the immunofluorescence.

### Protein expression and purification

All proteins were expressed in *E. coli* BL21 Rosetta (DE3) cells (catalog number: 70954, Sigma-Aldrich). For mCherry-PABPN1-WT/ΔRRM/R200A/ΔNter, bacteria were grown in lysogeny broth at 37°C and induced using 0.5 mM isopropyl-β-d-thiogalactopyranoside at 18°C for 16 hours until the optical density of a sample measured at a wavelength of 600 nm (optical density at 600 nm) = 0.6 to 0.8. The cells were resuspended in lysis buffer [150 mM NaCl, 50 mM tris-HCl (pH 7.5), 5 mM MgCl_2_, 4 mM dithiothreitol (DTT), 1 mM ethylenediaminetetraacetic acid, and protease inhibitor cocktail], and disrupted by means of sonication on ice, followed by centrifugation at 14,000 rpm for 1 hour at 4°C. mCherry-PABPN1-WT/ΔRRM/R200A/ΔNter were purified using a glutathione *S*-transferase column. Proteins were concentrated by means of centrifugal filtration in 150 mM NaCl, 50 mM tris-HCl (pH 7.5), 5 mM MgCl_2_, and 1 mM DTT. The proteins were flash-frozen and stored at −80°C. The concentrations of all proteins were estimated by measuring the absorbance at 280 nm using a spectrophotometer (NanoDrop).

### In vitro phase separation assays

In vitro PABPN1 phase separation was induced in a 20 mM tris-HCl buffer (pH 7.5) containing 130 mM KCl, 15 mM NaCl, and 1.5 mM MgCl_2_ (to stabilize the RNA secondary structure) along with 5 mM KH_2_PO_4_, BSA (1 mg/ml), and 10% polyethylene glycol, molecular weight 8000. Proteins were added as the last component to the sample, to induce uniform condensation. The protein concentration was 5.0 μM for PABPN1, unless mentioned otherwise. RNA diluted in RNase-free water was added at different concentrations, as indicated.

### Fluorescence recovery after photobleaching

FRAP was performed using an A1 microscope (Nikon) with a 60× oil objective. PABPN1 (in vivo) or PABPN1 condensates (in vitro) were bleached for 3 s using a fluorescence microscopy laser intensity of 70% at the wavelength 480 nm (for GFP) or 561 nm (for mCherry). Recovery was recorded immediately. The fluorescence intensity of the photobleached area was normalized to the intensity of the unbleached area.

### RNA sequencing library preparation

Oocytes were collected from the indicated genotypes (10 oocytes per sample). Each sample was directly lysed with 4.2 μl of lysis buffer [containing 0.2% Triton X-100, RNase inhibitor, deoxyribonucleotide triphosphate (dNTPs), oligo-dT primers, and 1:1000 ERCC spike-in] and immediately used for cDNA synthesis using the Smart-seq2 method, as described previously (*59*). Sequencing libraries were constructed from 500 pg of amplified cDNA using TruePrep DNA Library Prep Kit V2 for Illumina (catalog number: TD503, Vazyme, Nanjing, China) according to the manufacturer’s instructions. Barcoded libraries were pooled and sequenced on the HiSeq X Ten platform (Illumina, San Diego, CA, USA) with 150-bp paired-end reads.

### RNA sequencing analysis

RNA sequencing (RNA-seq) was performed using three biological replicates for all the samples. Raw reads were trimmed to 50 bp and mapped to the mouse genome (mm9) and ERCC spike-in sequences using Tophat v2.1.1. Guided by the reference annotation [the University of California Santa Cruz (UCSC) gene models], only uniquely mapped reads were assembled into transcripts using Cufflinks v2.2.1. The expression level of each transcript was quantified to the normalized FPKM and further normalized to the ERCC spike-in. Only transcripts with FPKM >1 in at least one sample were considered in all analyses. Functional annotation was performed using DAVID (https://david.ncifcrf.gov/tools.jsp). The Spearman correlation coefficient was calculated using the “cor” function. Other RNA-seq data used in this study are summarized in tables S3 to S5.

### RNA isolation and RT-qPCR

Collected oocytes (10 oocytes per sample) were directly lysed with 4.2 μl of lysis buffer (containing 0.2% Triton X-100, RNase inhibitor, dNTPs, and oligo-dT primers). cDNA was synthesized using the SuperScript II Reverse Transcription System (containing SuperScript II reverse transcriptase, SuperScript II first-strand buffer, RNase inhibitor, MgCl_2_, and betaine), as reported previously (*59*). RT-qPCR was performed using Power SYBR Green PCR Master Mix (Applied Biosystems, Life Technologies) and an Applied Biosystems 7500 Real-Time PCR System. Relative mRNA levels were normalized to the level of endogenous *Gapdh* mRNA (internal control). The relative transcription levels of the samples were compared with those of the control, followed by subsequent determination of the fold change. The RT-qPCR was performed in triplicate. The primer sequences used are listed in table S6.

### Method for detecting alternative splicing events

Alternative splicing was analyzed by CASH (comprehensive alternative splicing hunting) v2.2.1 software using UCSC reference genome (*60*). Alternative splicing events with delta_PSI > 0.1 and false discovery rate < 0.05 were considered credible and used for further analysis.

### Prediction of 3′UTR alternative polyadenylation

Dynamics analysis of alternative polyadenylation (APA) from RNA-seq data by DaPars v.2.0 program, which is freely available at https://github.com/3UTR/DaPars2 (*61*, *62*). Transcripts with 0.2 < PUI_Group_diff < −0.2 and P_val < 0.05 were considered credible and used for further analysis. The transcripts showed shift in APA are listed in table S7.

### Statistical analysis

Only littermates were used for WT control and knockout mice. Samples/organisms/participants of the same genotype/age were randomly allocated into experimental groups. The investigators were blinded to group allocation during oocyte manipulation and RNA-seq data collection and analysis. Oocytes with signs of degeneration before or after culture were excluded from the analyses. Statistical data have been presented as means ± SEM. Most experiments included at least three independent samples and were repeated at least three times. A two-tailed unpaired Student’s *t* test was used to compare the different groups; *P* < 0.05, *P* < 0.01, and *P* < 0.001 were considered statistically significant results and represented using asterisks (*), (**), and (***), respectively. “n.s.” indicates nonsignificant.

## Notes

View/request a protocol for this paper from *Bio-protocol*.
