# Supplementary material for: PABPN1 functions as a hub in the assembly of nuclear poly(A) domains that are essential for mouse oocyte development
Source: Sci Adv. 2022 Oct 28;8(43):eabn9016. doi: 10.1126/sciadv.abn9016 (PMC9616507; doi:10.1126/sciadv.abn9016)
Supplement: Supplementary file 1 — Figs. S1 to S8 Tables S1, S2, S4, and S6 [file sciadv.abn9016_sm.pdf]

Science Advances
AAAS

Supplementary Materials for

PABPN1 functions as a hub in the assembly of nuclear poly(A) domains that are essential for mouse oocyte development

Xing-Xing Dai *et al.*

Corresponding author: Heng-Yu Fan, hyfan@zju.edu.cn; Qian-Qian Sha, shaqianqian@zju.edu.cn

Sci. Adv. **8**, eabn9016 (2022)
DOI: 10.1126/sciadv.abn9016

The PDF file includes:

Figs. S1 to S8
Tables S1, S2, S4, and S6
Legends for tables S3, S5, and S7

Other Supplementary Material for this manuscript includes the following:

Tables S3, S5, and S7

Supplementary Figures

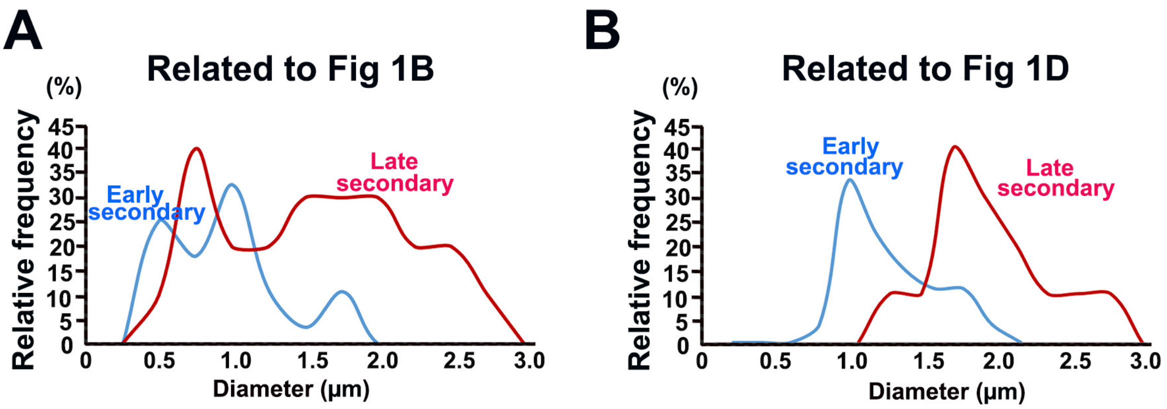

Fig. S1. The quantification of sizes of PA$^+$ and PABPN1 foci in growing oocytes.

A-B: Statistical analysis of the size of PA$^+$ (**A**) and PABPN1 (**B**) foci in Fig 1B and Fig1D.

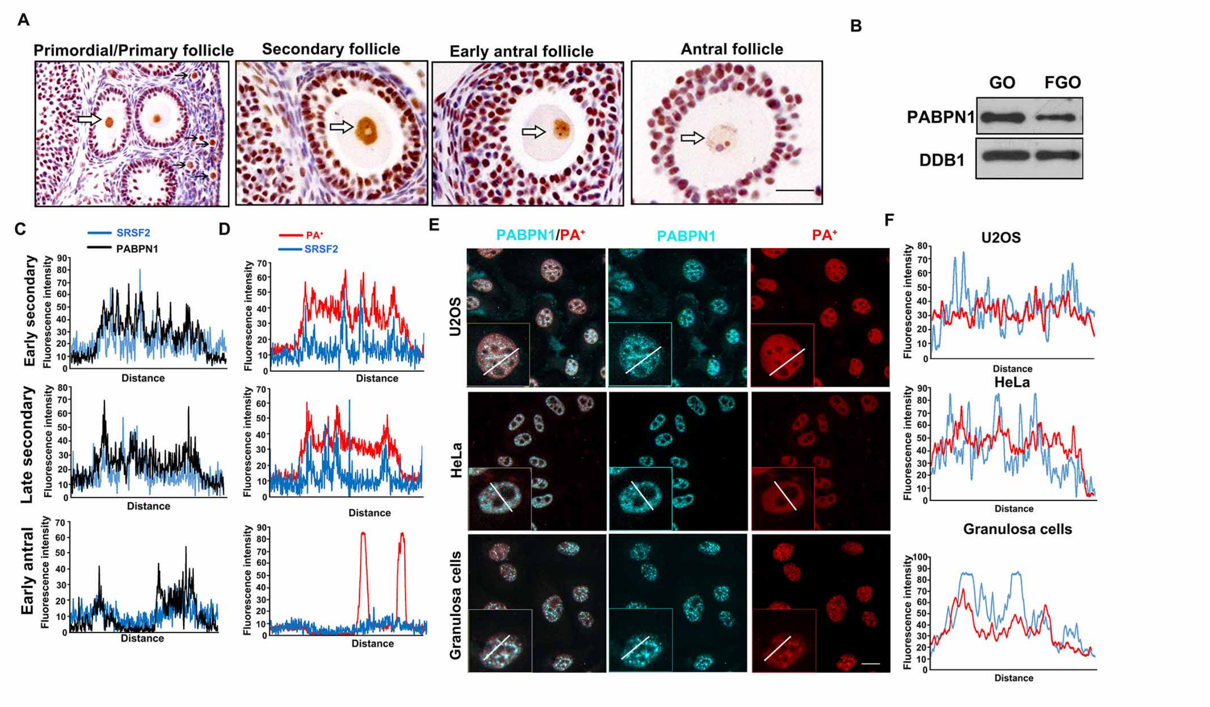

Fig. S2. The nuclear speckles marker SRSF2 co-localized with PABPN1 and PA$^+$ in growing oocytes.

A: Immunohistochemistry results showing the expression and localization of PABPN1 in oocytes from different follicular stages. The arrow indicates the location of PABPN1 in the nuclei of oocytes. Scale bar: 20 μm. **B:** Expression of PABPN1 in growing and fully grown mouse oocytes, as revealed using western blot analysis. DDB1 was used as the loading

control. **C:** ImageJ was used to quantitatively analyze the distribution of SC35 and PABPN1, indicated using a white dotted line. **D:** Quantitative analysis of the distribution of SC35 and PA^{+} (indicated using a white dotted line) using ImageJ. **E:** FISH and immunofluorescence to detect the distribution of PA^{+} and PABPN1 in cultured cells (U2OS, HeLa and granulosa cells). Scale bar: 20 μm. **F:** Quantitative analysis of the distribution of PABPN1 and PA^{+} in (**E**) using ImageJ software.

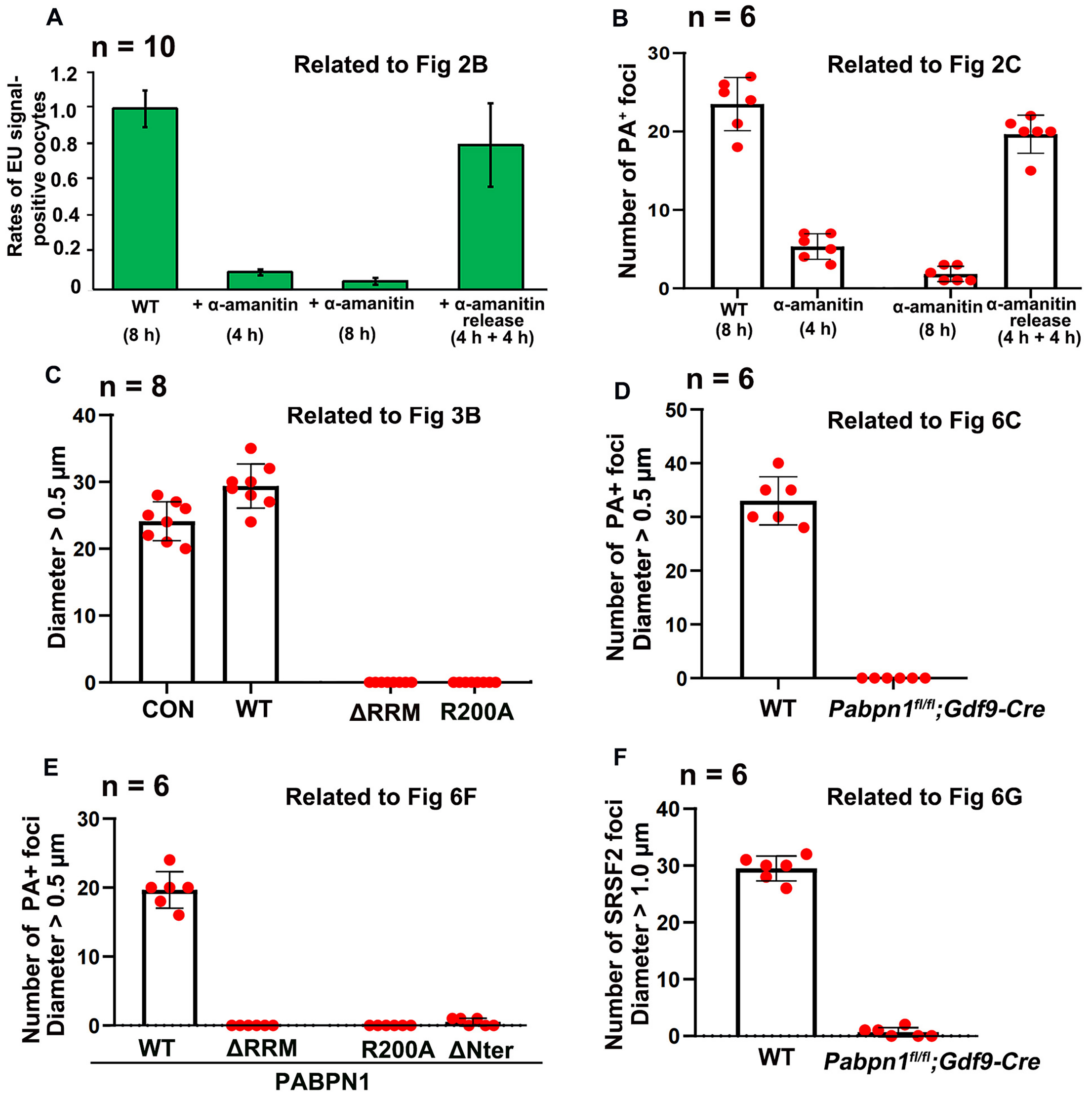

Fig. S3. The percentage of growing oocytes passed the criteria mentioned in Fig 2, Fig 3, and Fig 6.

A: The proportion of EU signal-positive growing oocytes before and after α-amanitin treatment in Fig 2B. **B:** The number of NPAD in oocytes with or without α-amanitin treatment

in Fig 2C. **C:** The number of NPADs with a diameter greater than 0.5 µm after overexpressing different forms of PABPN1 in Fig 3B. **D:** The number of PA^+ foci with a diameter greater than 0.5 µm in WT and *Pabpn1*-deletion oocytes in Fig 6C. **E:** The number of PA^+ foci with a diameter greater than 0.5 µm in *Pabpn1*-deleted oocytes after overexpressing different forms of PABPN1 in Fig 6F. **F:** The number of SRSF2 foci with a diameter greater than 1.0 µm in WT and *Pabpn1*-deleted oocytes in Fig 6G. Error bars represent the SEM.

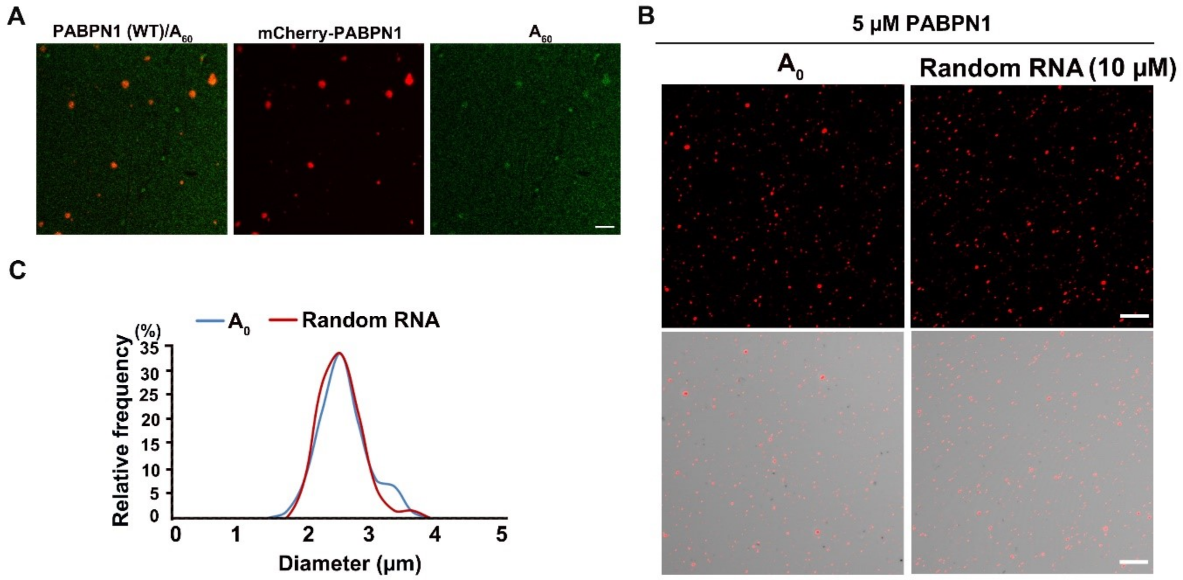

Fig. S4. The addition of random RNA did not promote further condensation of PABPN1. **A:** Representative images of co-localization of PABPN1 (5 µM) with FITC-A_{60}, on the basis of its condensation properties. Scale bar: 10 µm. **B:** Quantitative statistics of the size of the PABPN1 condensates in (**A**). **C:** *In vitro* phase separation assay of PABPN1 with or without random RNA. Scale bar: 10 µm.

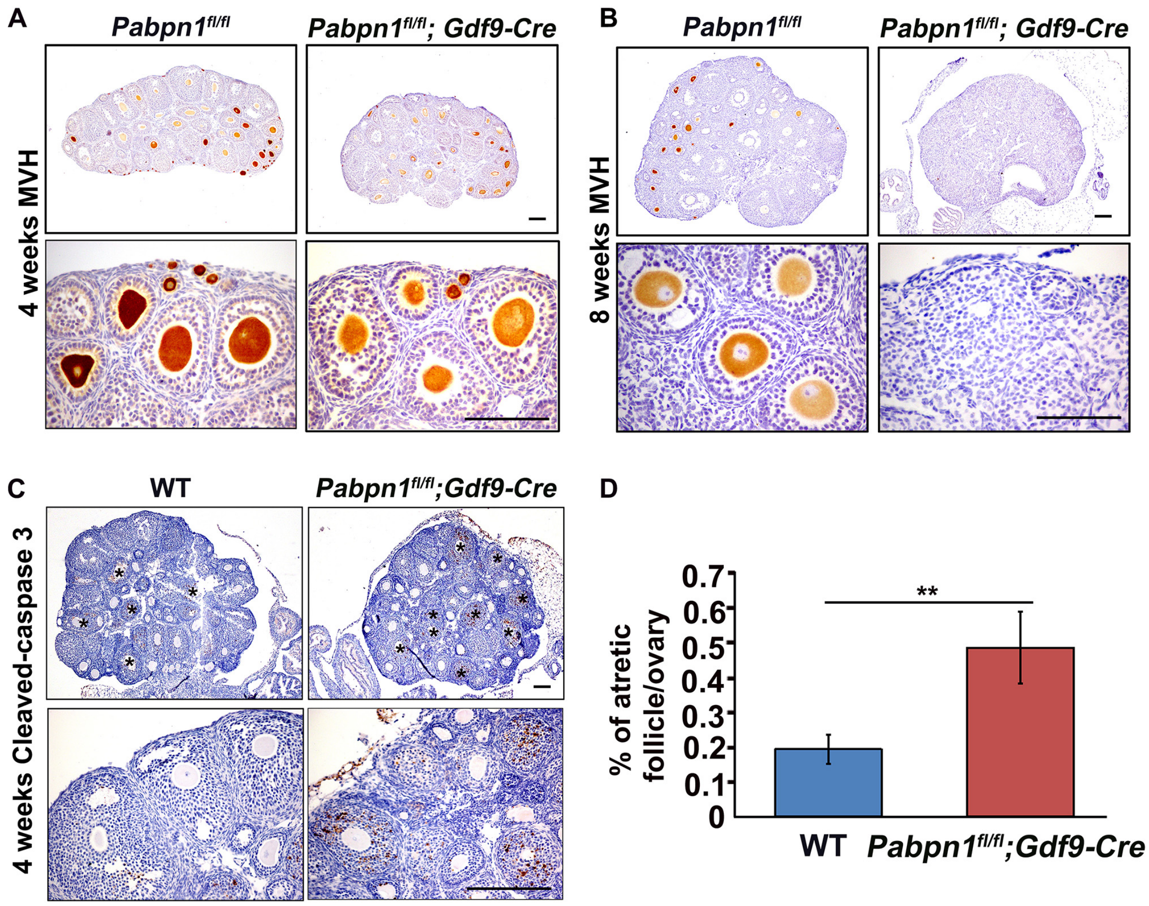

Fig. S5. Ovarian characteristics of WT and *Pabpn1$^{fl/fl}$;Gdf9-Cre* mice.

A-B: Immunohistochemistry results showed the expression of MVH in the ovaries of WT and *Pabpn1$^{fl/fl}$;Gdf9-Cre* mice at 4 and 8 weeks. Scale bar: 100 µm. **C:** Immunohistochemistry results showed the level of cleaved caspase 3 in the ovaries of WT and *Pabpn1$^{fl/fl}$;Gdf9-Cre* mice at 4 weeks of age. Asterisks indicate atretic follicles. Scale bar: 100 µm. **D:** Quantification results for cleaved caspase 3-positive (atretic) follicles in ovaries of 4-week-old WT and *Pabpn1$^{fl/fl}$;Gdf9-Cre* female mice. Error bars represent the SEM. Statistical analysis was performed using Student's *t*-test. $^{**}P < 0.01$.

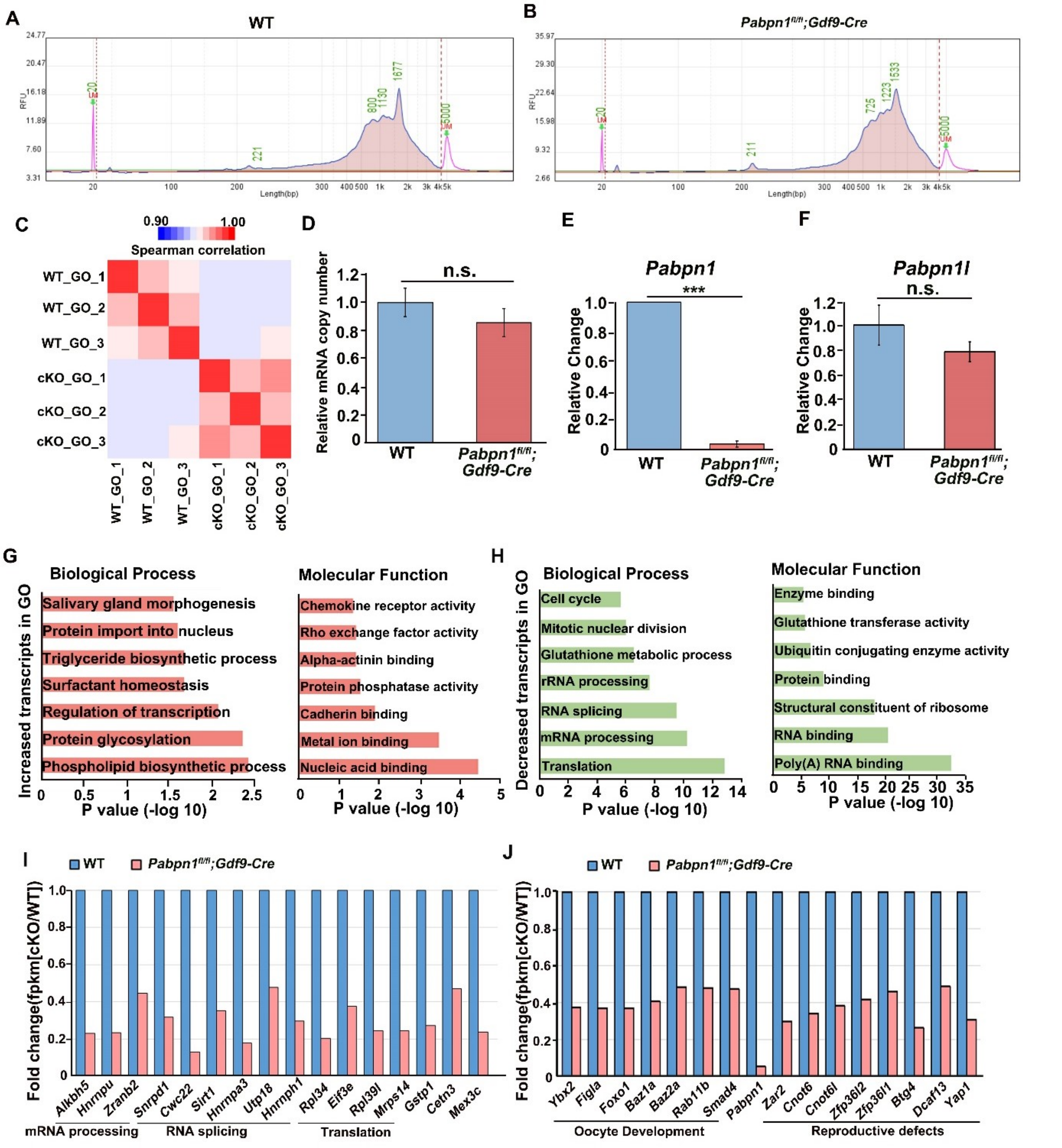

Fig. S6. Analysis of transcriptome of oocytes from *Pabpn1$^{fl/fl}$;Gdf9-Cre* and WT mice.

A-B: Global cDNA length distribution map from WT and *Pabpn1$^{fl/fl}$;Gdf9-Cre* mouse oocytes before RNA sequencing. **C:** Heatmap of Spearman's correlation coefficients of the total transcripts between WT and *Pabpn1*-null growing oocytes. **D:** Relative mRNA copy numbers in WT and *Pabpn1*-null growing oocytes. Error bars indicate the values of three biological replicates. **E-F**: qRT-PCR results showing the relative expression levels of *Pabpn1* and *Pabpn1l* in oocytes collected from WT and *Pabpn1$^{fl/fl}$;Gdf9-Cre* mice. Error bars represent the SEM. ***P < 0.001 as assessed using two-tailed Student's t-test. **G:** GO analysis of

transcripts whose levels increased in *Pabpn1*$^{fl/fl}$*;Gdf9-Cre* mice, as compared to those in WT mice. **H:** GO analysis of transcripts whose levels decreased in *Pabpn1*$^{fl/fl}$*;Gdf9-Cre* mice, as compared to those in WT mice. **I-J:** RNA-seq results showing the relative expression levels of representative transcripts in WT and *Pabpn1*$^{fl/fl}$*;Gdf9-Cre* mouse oocytes.

A

Gene	SplicingType
Alg13	AltEnd
Rdx	AltEnd
Fbxw28	AltEnd
Zfp655	AltEnd
Txnl4a	AltEnd
Trim60	AltEnd
Polr1d	AltEnd
Tmt1	IR
Nlrp14	IR
Ikbkb	IR
Fam221a	Cassette
Oas1h	Cassette_multi

B

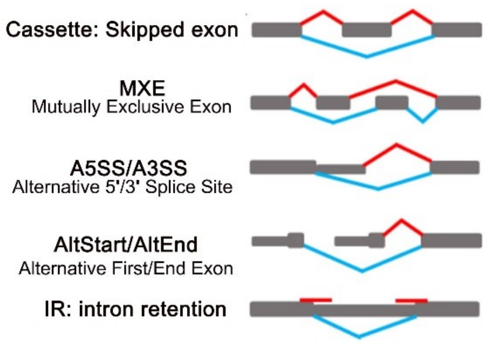

C

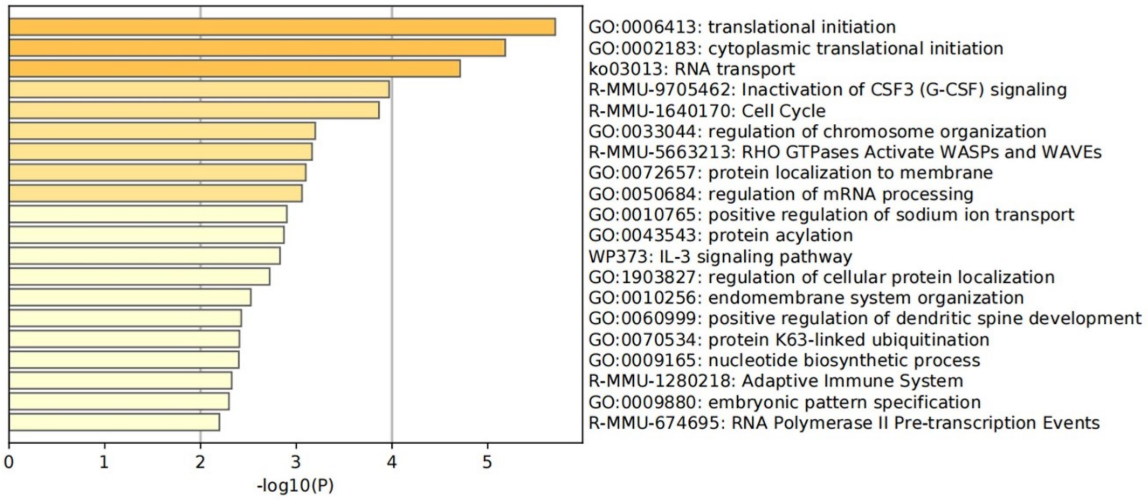

Fig. S7. *Pabpn1* deletion has little effect on the intron splicing of transcripts in oocytes.

A: Transcripts with abnormal intron splicing in *Pabpn1*-deletion oocytes, as compared to those in WT oocytes. **B:** Schematic diagram showing several types of intron-splicing abnormalities. **C:** GO analysis of transcripts with 3′-UTR shortening in *Pabpn1*$^{fl/fl}$*;Gdf9-Cre* mice oocytes, as compared to those in WT oocytes.

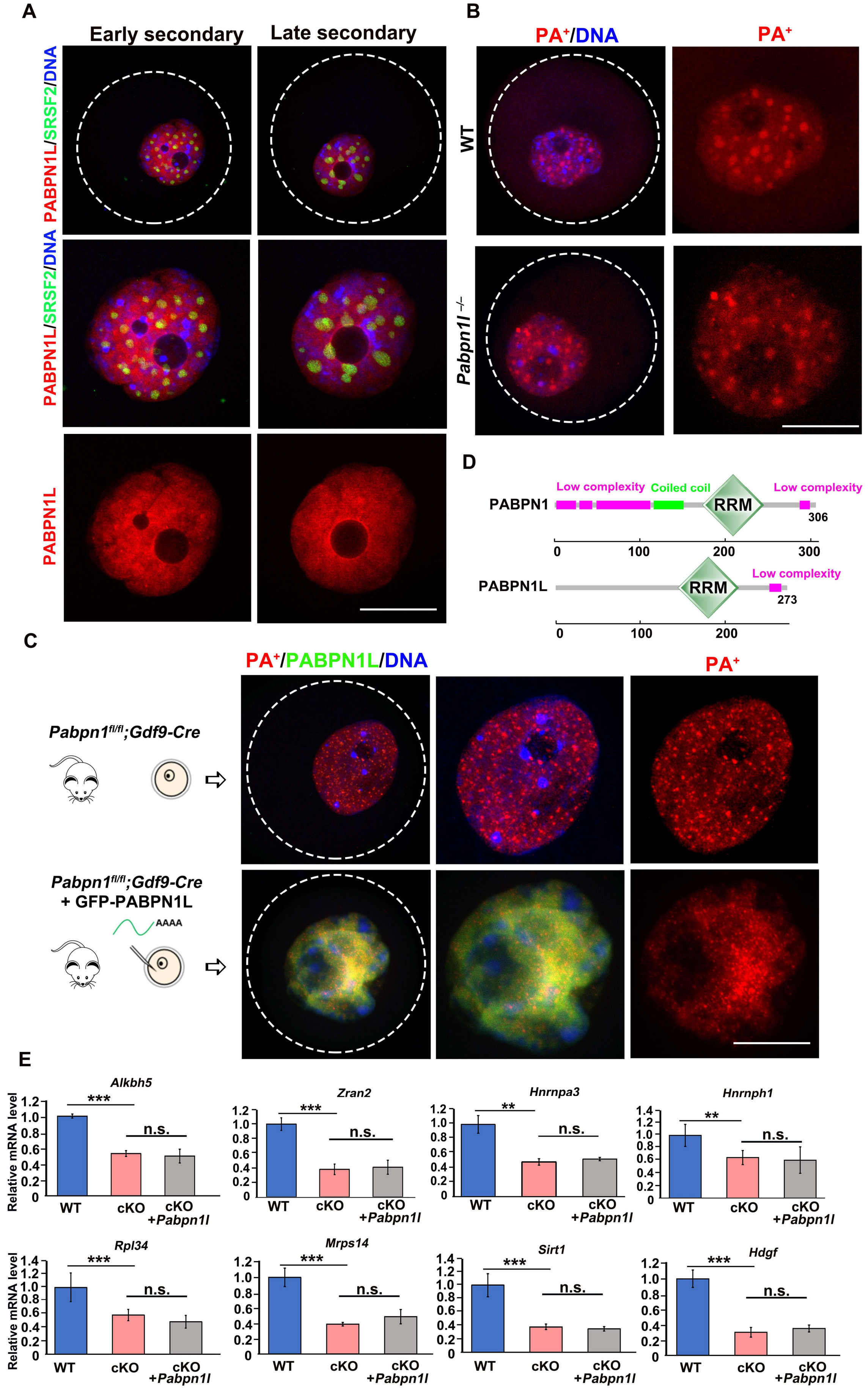

A
Early secondary
Late secondary
PABPN1L/SRSF2/DNA
PABPN1L/SRSF2/DNA
PABPN1L
B
PA+/DNA
PA+
WT
Pabpn1l −/−
D
PABPN1
Low complexity
Coiled coil
RRM
Low complexity
306
0
100
200
300
PABPN1L
RRM
Low complexity
273
0
100
200
C
PA+/PABPN1L/DNA
PA+
Pabpn1fl/fl;Gdf9-Cre
Pabpn1fl/fl;Gdf9-Cre
+ GFP-PABPN1L
AAAA
E
Alkbh5
Zran2
Hnrnpa3
Hnrnph1
Relative mRNA level
***
**
n.s.
WT
cKO
cKO +Pabpn1l
Rpl34
Mrps14
Sirt1
Hdgf

Fig. S8. PABPN1L could not rescue the defects in *Pabpn1* knockout oocytes.

A: Immunofluorescence results showed the distribution of PABPN1L and SRSF2 in wild-type growing oocytes. Scale bars, 20 μm. **B:** Immunofluorescence and FISH results showed the distribution of PA^{+} in WT and *Pabpn1l* knockout oocytes. Scale bars, 20 μm. **C:** The FISH results showed the distribution of PA^{+} when the *Pabpn1* knockout oocytes were microinjected with mRNAs encoding PABPN1L. Scale bars, 20 μm. **D:** The diagram shows the prediction of the domains in PABPN1 and PABPN1L, respectively. The protein domain prediction website is: http://smart.embl-heidelberg.de/. **E:** Quantitative RT-PCR results showing the relative levels of the indicated transcripts in WT and *Pabpn1*-deleted oocytes with or without PABPN1L overexpression. Error bars represent the SEM. Statistical analysis was performed using Student's *t*-test. $^{**}P < 0.01$, $^{***}P < 0.001$. n.s.: non-significant.

Supplementary Tables

Table S1. Antibody information.

**Protein name**	**Manufacture (catalogue number)**	**Applications (working dilution)**	**RRID**
**PABPN1**	Bethy (A303-523A)	WB (1:500)<br>IF (1:200)<br>IHC (1:200)	AB_10953822
**SRSF2**	Sigma (SAB4200725)	IF (1:200)	NA
**MVH**	abcom (ab27591)	IHC (1:200)	AB_11139638
**CPSF4**	Proteintech (15023-1-AP)	IF (1:200)	AB_2084544
**DDB1**	Epitomics (3821-1)	WB (1:1000)	AB_10896720
**PABPN1L**	Made by company	IF (1:200)	NA
**HA**	Cell Signaling Technology (3724)	IF (1:400)	AB_1549585
**Cleaved Caspase 3**	Cell Signaling Technology (9669)	IHC (1:200)	AB_2069869

Table S2. Spearman correlation coefficients among WT and *Pabpn1*-null growing oocytes.

		**WT**			***Pabpn1*$^{fl/fl}$*;Gdf9-Cre* mice**		
	**Sample**	**1**	**2**	**3**	**1**	**2**	**3**
**WT**	**1**	1.00	0.97	0.96	0.95	0.95	0.95
	**2**	0.97	1.00	0.96	0.95	0.94	0.95
	**3**	0.96	0.96	1.00	0.95	0.95	0.95
Pabpn1*$^{fl/fl}$*; Gdf9-Cre	**1**	0.95	0.95	0.95	1.00	0.97	0.97
	**2**	0.95	0.94	0.95	0.97	1.00	0.97
	**3**	0.95	0.95	0.95	0.97	0.97	1.00

Table S3. FPKMs of transcripts decreased or increased for more than 2 folds in oocytes from WT and *Pabpn1*$^{fl/fl}$*;Gdf9-Cre* mice. (In a separate xlsx file).

Table S4. Quality control of RNA-seq results (growing oocytes from WT and *Pabpn1*$^{fl/fl}$*;Gdf9-Cre* mice).

**Sample**	**Total reads**	**Mapping efficiency**
WT-GO-Rep1	13,371,275	87.6%
WT-GO-Rep2	18,295,533	87.9%
WT-GO-Rep3	21,920,467	89.9%
Pabpn1-GO-Rep1	19,771,960	88.3%
Pabpn1-GO-Rep2	18,326,856	90.1%
Pabpn1-GO-Rep3	17,821,766	88.9%

Table S5. FPKMs of RNA-seq results (in a separate xlsx file).

Table S6. Primer sequences.

Primer name	Genes targeted	Application	Sequences (5′-3′)
Alkbh5-CDS-qt-F	*Alkbh5*	Real-time PCR	5′- CGCGGTCATCAACGACTACC -3′
Alkbh5-CDS-qt-R	*Alkbh5*	Real-time PCR	5′- ATGGGCTTGAACTGGAACTTG -3′
Zran2-CDS-qt-F	*Zran2*	Real-time PCR	5′- GAATTTCCGAGTCAGTGACGG -3′
Zran2-CDS-qt-R	*Zran2*	Real-time PCR	5′- CCCCAGCTTTCATCATCTTGG -3′
Hnrnpa3-CDS-qt-F	*Hnrnpa3*	Real-time PCR	5′- GAGGAGGGCCATGATCCAAAG -3′
Hnrnpa3-CDS-qt-R	*Hnrnpa3*	Real-time PCR	5′- GTCTGTAAGTGTGCCCCATTT -3′
Hnrnph1-CDS-qt-F	*Hnrnph1*	Real-time PCR	5′- AAATGGGGCTCAAGGTATTCG -3′
Hnrnph1-CDS-qt-R	*Hnrnph1*	Real-time PCR	5′- GGACCAGTATGCTTCAACACC -3′
Rpl34-CDS-qt-F	*Rpl34*	Real-time PCR	5′- TCCAGCGTTTGACATACCGC -3′
Rpl34-CDS-qt-R	*Rpl34*	Real-time PCR	5′- TAGGTGCTTTCCCAACCTTCT -3′
Mrps14-CDS-qt-F	*Mrps14*	Real-time PCR	5′- CTCCATCAGCGTCAGGTCAAG -3′
Mrps14-CDS-qt-R	*Mrps14*	Real-time PCR	5′- CGAAGCCTCTCATCTGCATATT -3′
Sirt1-CDS-qt-F	*Sirt1*	Real-time PCR	5′- GCTGACGACTTCGACGACG -3′
Sirt1-CDS-qt-R	*Sirt1*	Real-time PCR	5′- TCGGTCAACAGGAGGTTGTCT -3′
Hdgf-CDS-qt-F	*Hdgf*	Real-time PCR	5′- AGGGCAGCAGCGACGAAGAA -3′
Hdgf-CDS-qt-R	*Hdgf*	Real-time PCR	5′- TCTCACCCTCCAAGGCAGCT -3′
Mex3c-CDS-qt-F	*Mex3c*	Real-time PCR	5′- TGTCACTGGACGGAAAGAAGA -3′

Mex3c-CDS-qt-R	*Mex3c*	Real-time PCR	5′- TGCACGAATCATAGAAAAGTGCT -3′
Alkbh5-PAT-F	*Alkbh5*	PAT assay	5′- CTATGCCCCAGCAGCTCATTGC -3′
Zranb2-PAT-F	*Zranb2*	PAT assay	5′- TCGACTTTCTGAGTTGCCCAG -3′
Sirt1-PAT-F	*Sirt1*	PAT assay	5′- GTAATGTGAGGAGTCAGCACCG -3′
Btg4-PAT-F	*Btg4*	PAT assay	5′- GTAGGTTTTCAACTAAGGAAGAT -3′
Rpl39l-PAT-F	*Rpl39l*	PAT assay	5′- TCCACAATGGATTCAGATGA -3′
PAT-R	N.A.	Anchor primer for PAT assay	5′- GCGAGCTCCGCGGCCGCGTTTTTTTTTTTT -3′
Ccnb1-CDS-qt-F	*Ccnb1*	Real-time PCR	5′- TCGAGCAGCACACTTTGGCC -3′
Ccnb1-CDS-qt-R	*Ccnb1*	Real-time PCR	5′- TCAGCGCTAAGCAGAAAGC -3′
Ccnb1-prox-qt-F	*Ccnb1*	Real-time PCR	5′- CTCCAATAGACTGCTACATC -3′
Ccnb1-prox-qt-R	*Ccnb1*	Real-time PCR	5′- GAAGAGCAAGTAAACACGGT -3′
Ccnb1-dist-qt-F	*Ccnb1*	Real-time PCR	5′- TGGCTTCATTCATAGTAGCTC -3′
Ccnb1-dist-qt-R	*Ccnb1*	Real-time PCR	5′- CAGCAAATGAGACAGGATTC -3′
Yipf4-CDS-qt-F	*Yipf4*	Real-time PCR	5′- GTCTCATGGATTATAACCAT -3′
Yipf4-CDS-qt-R	*Yipf4*	Real-time PCR	5′- GAATATCCTATAACTCCAAG -3′
Yipf4-prox-qt-F	*Yipf4*	Real-time PCR	5′- AGATAGAGAGGCAGGCTTAAAGTC -3′
Yipf4-prox-qt-R	*Yipf4*	Real-time PCR	5′- CAGAATTCAGAATTCTGACC -3′
Yipf4-dist-qt-F	*Yipf4*	Real-time PCR	5′- CTAATAGTTGCTGGTATAGA -3′
Yipf4-dist-qt-R	*Yipf4*	Real-time PCR	5′- ATCTCTTGCTCCTTTGGTCA -3′
Snx16-CDS-qt-F	*Snx16*	Real-time PCR	5′- ATTAAAAGAGATGTTTCCAGGC -3′
Snx16-CDS-qt-R	*Snx16*	Real-time PCR	5′- TGAAGAAATGCTTGTAGTCC -3′
Snx16-prox-qt-F	*Snx16*	Real-time PCR	5′- TTGTTATCAGGGCCCATATT -3′
Snx16-prox-qt-R	*Snx16*	Real-time PCR	5′- GACAAAGCAAGCACACATAT -3′
Snx16-dist-qt-F	*Snx16*	Real-time PCR	5′- AATGCCTTGTCTCTTCTTGA -3′
Snx16-dist-qt-R	*Snx16*	Real-time PCR	5′- CCAACTTGAACTTCAAGATG -3′
Tmed2-CDS-qt-	*Tmed2*	Real-time PCR	5′- GCCCACATGGATGGGACATACAAGT -3′

F			
Tmed2-CDS-qt-R	*Tmed2*	Real-time PCR	5′- TTGTCCTTTGGGAGCCTCCC -3′
Tmed2-prox-qt-F	*Tmed2*	Real-time PCR	5′- TCTGAACGTTCATGACTTATGT -3′
Tmed2-prox-qt-R	*Tmed2*	Real-time PCR	5′- CAAGTAAGAATTATAGGCCA -3′
Tmed2-dist-qt-F	*Tmed2*	Real-time PCR	5′- AGGTTTTCATGTTCAGGACA -3′
Tmed2-dist-qt-R	*Tmed2*	Real-time PCR	5′- CTGAAGTGTTTTCACACACA -3′

Table S7. Transcripts showed shift in APA in *Pabpn1*-deletion samples versus control samples. (In a separate xlsx file).
